# Single-cell BCR and transcriptome analysis after influenza infection reveals spatiotemporal dynamics of antigen-specific B cells

**DOI:** 10.1016/j.celrep.2021.109286

**Published:** 2021-06-22

**Authors:** Nimitha R. Mathew, Jayalal K. Jayanthan, Ilya V. Smirnov, Jonathan L. Robinson, Hannes Axelsson, Sravya S. Nakka, Aikaterini Emmanouilidi, Paulo Czarnewski, William T. Yewdell, Karin Schön, Cristina Lebrero-Fernández, Valentina Bernasconi, William Rodin, Ali M. Harandi, Nils Lycke, Nicholas Borcherding, Jonathan W. Yewdell, Victor Greiff, Mats Bemark, Davide Angeletti

**Affiliations:** 1 Department of Microbiology and Immunology, Institute of Biomedicine, University of Gothenburg, Gothenburg, Sweden; 2 Department of Biology and Biological Engineering, National Bioinformatics Infrastructure Sweden, Science for Life Laboratory, Chalmers University of Technology, Göteborg, Sweden; 3 Department of Biochemistry and Biophysics, National Bioinformatics Infrastructure Sweden, Science for Life Laboratory, Stockholm University, Solna, Sweden; 4 Immunology Program, Memorial Sloan Kettering Cancer Center, New York, NY, USA; 5 Vaccine Evaluation Center, BC Children’s Hospital Research Institute, University of British Columbia, Vancouver, BC, Canada; 6 Department of Pathology and Immunology, Washington University, St. Louis, MO, USA; 7 Laboratory of Viral Diseases, National Institutes of Allergy and Infectious Diseases, National Institutes of Health, Bethesda, MD, USA; 8 Department of Immunology, University of Oslo, Oslo, Norway; 9 Region Västra Götaland, Department of Clinical Immunology and Transfusion Medicine, Sahlgrenska University Hospital, Gothenburg, Sweden

## Abstract

B cell responses are critical for antiviral immunity. However, a comprehensive picture of antigen-specific B cell differentiation, clonal proliferation, and dynamics in different organs after infection is lacking. Here, by combining single-cell RNA and B cell receptor (BCR) sequencing of antigen-specific cells in lymph nodes, spleen, and lungs after influenza infection in mice, we identify several germinal center (GC) B cell subpopulations and organ-specific differences that persist over the course of the response. We discover transcriptional differences between memory cells in lungs and lymphoid organs and organ-restricted clonal expansion. Remarkably, we find significant clonal overlap between GC-derived memory and plasma cells. By combining BCR-mutational analyses with monoclonal antibody (mAb) expression and affinity measurements, we find that memory B cells are highly diverse and can be selected from both low- and high-affinity precursors. By linking antigen recognition with transcriptional programming, clonal proliferation, and differentiation, these finding provide important advances in our understanding of antiviral immunity.

## Graphical abstract

**Graphical abstract:**
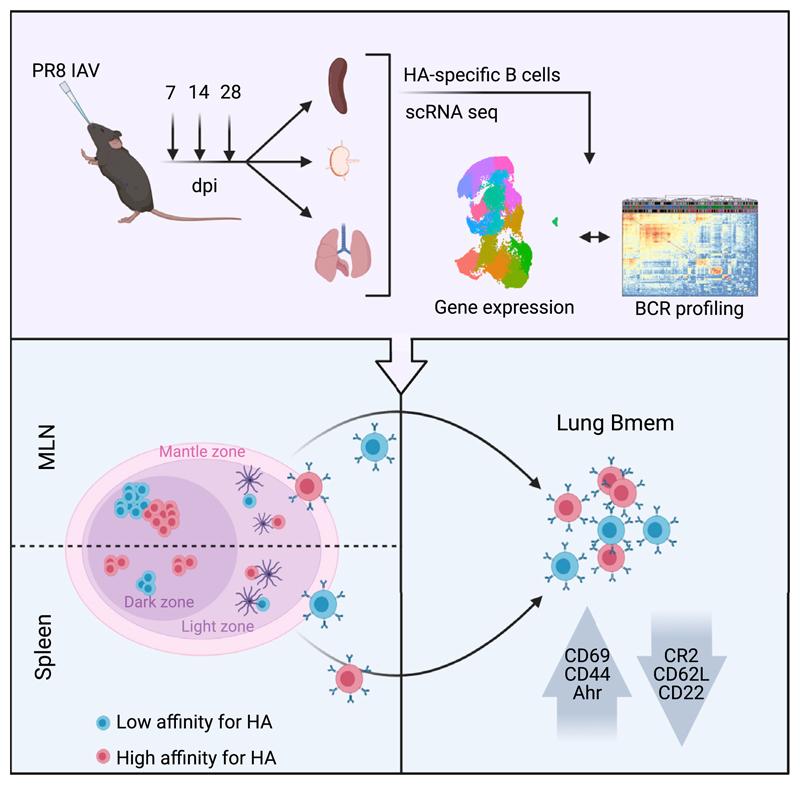

## Introduction

Viral respiratory infections caused by influenza-, orthopneumo-, or corona-virus are major concerns worldwide. Influenza A virus (IAV) is a highly prevalent, respiratory virus that causes significant morbidity and mortality in humans (Iuliano et al., 2018). B-cell-derived antibodies (Abs) are a central feature of adaptive immunity to viruses. Abs can greatly reduce viral pathogenicity in primary infections and can provide complete protection against disease-causing reinfections (Lam and Baumgarth, 2019). In mice, intranasal (i.n.) infection with IAV initiates B cell responses in several organs, characterized by a robust, early extrafollicular plasmablast (PB) response, followed by persistent germinal center (GC) formation in the draining mediastinal lymph nodes (mlns) and diffuse memory B cell (Bmem) dispersion across several organs (Angeletti et al., 2017; Boyden et al., 2012; Frank et al., 2015; Jooet al., 2008; Rothaeusler and Baumgarth, 2010). Respiratory virus infections can also promote circulating blood cells to generate inducible bronchus-associated lymphoid tissues (iBALTs) in the lung parenchyma (Moyron-Quiroz et al., 2004), resulting in the formation of GC-like structures in mouse lungs by 14 days post infection (dpi) with IAV (Denton et al., 2019; Tan et al., 2019).

The viral surface-glycoprotein hemagglutinin (HA) is the immunodominant target of B cell response to IAV infection and immunization (Altman et al., 2015; Angeletti and Yewdell, 2018). Nevertheless, comprehensive studies assessing the link between transcriptional status and the clonal diversity of B cell populations at different developmental stages within or between organs after respiratory viral infections are lacking. Deciphering how B cell receptor (BCR) characteristics are linked to cell differentiation is crucial for our ability to understand and ultimately manipulate B cell responses with more-effective vaccines or adjuvants.

Few studies have identified lung Bmems as critical in preventing IAV reinfection (Allie et al., 2019; Onodera et al., 2012). These tissue-resident Bmems (Allie et al., 2019) appear to have broader specificity than splenic Bmems do (Adachi et al., 2015). However, virtually nothing is known about the transcriptional programming leading to their formation, their BCR profile, and whether they originate from lung-iBALT versus other lymphoid organs. Better appreciation of the origin and formation of lung-resident memory cells after infection is a crucial first step in developing mucosal vaccines against respiratory viruses.

GCs form as a consequence of rapid clonal proliferation during T-cell-dependent B cell responses and are the site of B cell affinity maturation through selection of high-affinity clones generated via somatic hypermutation (SHM) (Mesin et al., 2016). Signals that regulate terminal B cell differentiation to PBs or Bmems have primarily been studied using model antigens and transgenic mice (Kräutler et al., 2017; Phan et al., 2006; Shinnakasu et al., 2016; Smith et al., 1997; Suan et al., 2017a, 2017b; Weisel et al., 2016). The general consensus is that B cells with higher avidity to antigens will differentiate into PBs, whereas B cells of lower avidity will become Bmems (Viant et al., 2020; Shinnakasu et al., 2016; Suan et al., 2017a, 2017b). In addition, a temporal switch, with Bmems being produced only early in the anti-(4-hydroxy-3-nitrophenyl)acetyl (anti-NP) response was identified (Weisel et al., 2016). Importantly, unlike most natural responses, both NP and hen egg lysozyme (HEL) models require only a single mutation for the germline V region in the BCR to mature from low to high avidity. Whether a similar selection of lower-avidity GC cells into the memory compartment occurs after viral infection and how the selection differs among organs is unclear. A recent study (Wong et al., 2020) suggested that such affinity selection might not be that pronounced during flavivirus infection and that Bmems in the spleen might arise from lower-affinity germline precursors. This is particularly relevant in understanding how the first encounter with a virus shapes Bmem formation, a central feature of the original antigenic-sin phenomenon in anti-IAV responses (Henry et al., 2018; Yewdell and Santos, 2021).

To address these questions and to overcome limitations in previous studies, we sequenced single-HA-specific B cells to correlate their transcriptome with their paired heavy and light chain BCRs from different organs and across time points post-i.n. IAV infection. Our data provide important mechanistic insights into B cell differentiation upon respiratory viral infection.

## Results

### scRNA-seq of antigen-specific B cells after influenza infection identifies a range of B cell differentiation stages

**Figure 1:**
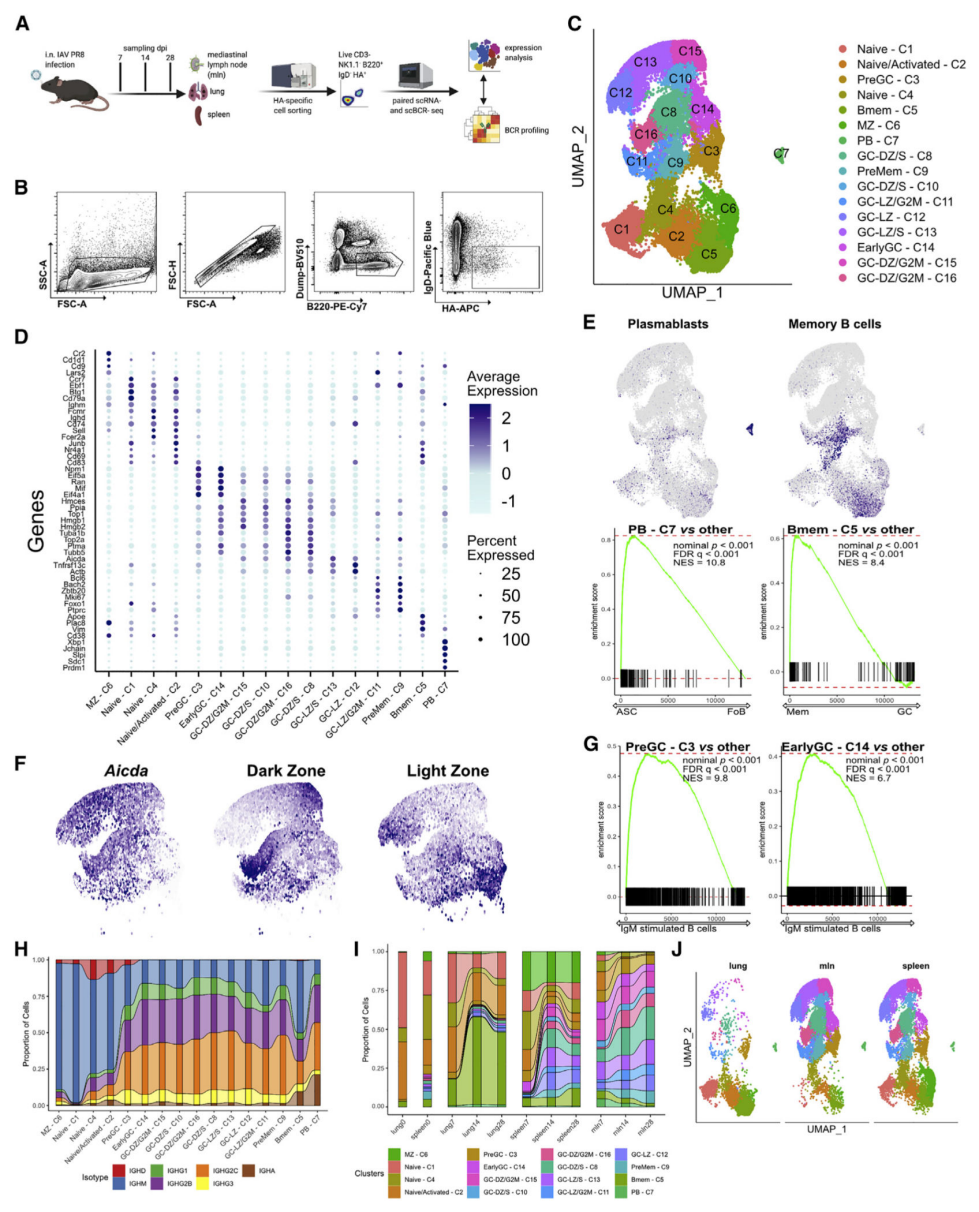
Dynamics of antiviral B cell response at single-cell resolution is organ specific (A) Schematic diagram of the experimental setup of influenza infection, cell sorting, followed by scRNA-seq and BCR profiling. (B) Representative gating for the cell sorting of single HA^+^ IgD^−^ B cells. (C) UMAP plot of unsupervised clustering of HA-specific B cells, combining all organs and dpi. (D) Mean expression of the top-five marker genes for each cell cluster. Color intensity denotes average expression, whereas dot size is the percentage of cells expressing the gene. (E) On the top UMAP plot, as in (C), showing average expression of gene signatures associated with plasma blasts and memory B cell programs from Bhattacharya et al. (2007). On the bottom, enrichment score from GSEA comparing PB-C7s to all other clusters for antibody-secreting cell (ASC) genes versus follicular B cell (FoB) genes from Shi et al. (2015) and Bmem-C5s to all other clusters for memory genes versus GC genes from Laidlaw et al. (2020). (F) UMAP plot of GC clusters showing average expression of *Aicda* and gene signatures associated with dark and light zone programs from Victora et al. (2010). (G) Enrichment score from GSEA comparing PreGC-C3 and EarlyGC-C14 to all others for genes involved in B cell activation and differentiation from Fowler et al. (2015). (H) Alluvial plot showing proportion of cells with defined antibody isotype for each cluster. (I) Alluvial plot showing proportion of cells for each UMAP cluster, as in (C), divided by organ and dpi. (J) UMAP plot of infected mice divided by organ.

i.n. mouse infection with IAV is a well-established, acute respiratory viral infection model. We infected mice i.n. with IAV PR8 and tracked antigen-specific responses at 7, 14, and 28 dpi by sorting antigen experienced, HA-binding immunoglobulin D (IgD^−^) B cells from individual lungs, spleen, and mlns (Figures 1A, 1B, and S1A). As a control, total B cells (50% of cells based on live B220^+^ IgD^+/−^ and 50% of cells based on live B220^+^ IgD^+/−^) were also sorted from spleen and lungs of two mice (Figure S1B). We subjected antigen-specific cells to single-cell RNA sequencing (scRNA-seq) paired with single-cell B cell receptor sequencing (scBCR-seq). In total, we analyzed results from 8,722 cells from two naive mice and 30,242 cells from eight infected mice (1,878, 15,428, and 12,936 cells, respectively, from 7, 14, and 28 dpi).

Unsupervised clustering, using the Sauron implementation of the Seurat package, distinguished 16 populations of HA-specific B cells that clustered according to their transcriptional profile (Figure 1C). Differential gene expression analysis allowed us to define specific cell populations (Figure 1D). Cell populations were defined using genes canonical for specific differentiation states, including *Ighd, Aicda, Bcl6, Mki67, Cd83, Cd38, Cxcr4, Ly6d, Cd1d1, Foxo1*, *Ccr6, Irf4, Sdc1*, and *Prdm1.* Marginal zone (MZ) B cells (cluster C6) were characterized by landmark genes *Cd1d1* and *Cd9.* Clusters C1, C4, and C2 comprised a mix of naive and activated cells expressing Ccr7, *Ebf1, Cd74,* and *Nr4a1* (Nur77). It is possible that these cells were also partially activated *in vitro* by binding to HA. In naive mice, 35%, 74%, and 74% of cells in C1, C2, and C4 expressed *Ighd,* respectively, and those clusters were overrepresented (Figure S1B). *Ighm* was particularly highly expressed in C1. C7 was high in canonical PB markers *Irf4, Slpi, Sdc1,* and *Prdm1* and was confirmed by previously described gene signatures (Figure 1E) (Bhattacharya et al., 2007; Shi et al., 2015). In C5, Bmem-signature genes were highly expressed (Bhattacharya et al., 2007), and the nature of that population was further strengthened using gene sets from Laidlaw et al. (2020) (Figure 1E). Interestingly, GC cluster C9 also showed high expression of genes associated with Bmem fate (see below for further discussion). Finally, clusters C3, C8, and C9–16 all expressed prototypical GC signature genes (Victora et al., 2010, 2012). We could readily divide GC clusters into light zone (LZ) and dark zone (DZ) cells using sets of genes known to distinguish these subsets (Figure 1F) (Victora et al., 2010). Despite having regressed out cell cycle influence, GC clusters still separated based on cell cycle phase, as expected (Figure S1C). We identified two LZ clusters (C3 and C14) that expressed gene signatures typical for initiation of signaling cascades and Gene Set Enrichment Analysis (GSEA) confirmed signatures consistent with recently antigen-activated B cells (Figure 1G) (Fowler et al., 2015).

Indeed, cluster C3 cells were mostly in G1 phase (PreGC), whereas C14 cells were entering the cell cycle (earlyGC). Clusters C15, C10, C16, and C8 had similar signatures, indicative of DZ GC B cells, with only differences in cell cycle status, with C16 in G2M, C8 and C10 in S phase, and C15 between G2M and G1, indicating cells exiting the cell cycle. Cluster C12 had a strong LZ signature, as did C11, which was split between G2M and G1 and C13, in the S phase (Figures S1B and S1C). Comparison of GC clusters using signatures derived from human tonsils (Holmes et al., 2020), confirmed our clusters assignments (Figure S1D).

To incorporate BCR sequence data into the overall analysis, we compiled the sequence data, isotype, and somatic mutations for the heavy-chain sequences using the Immcantation pipeline (Gupta et al., 2015; Vander Heiden et al., 2014) and scRepertoire (Borcherding et al., 2020) to define clonal status and expansion. Consistent with the recent demonstration of pre-GC class switching (King et al., 2021; Roco et al., 2019), cluster preGC C3 exhibited more than 60% class-switched BCR sequences (Figure 1H) and was enriched for the class-switch recombination signature (King et al., 2021) (Figures S1E and S1F). Therefore, we identified PreGC-C3 cells as those actively recruited into the earlyGC C14. Supporting this hypothesis, the fraction of B cells within these two clusters was almost twice as many at day 7 compared with days 14 and 28. The continued presence of these clusters weeks after infection is consistent with continued replenishment of the GC reaction (Figure 1I). Approximately one half of the HA-Bmem cells (cluster C5) had class switched, whereas all GC cluster cells were dominated by IgG2b/c. IgA BCRs were highly enriched both in HA-Bmems and in PBs in all organs, suggesting a preferential recruitment of IgA cells (Figures 1I and S1G).

### Antigen-specific clusters are organ specific and independent of days after infection

Although most clusters were found in all organs, surprisingly, HA^+^ B cell clusters were largely specific to organ and not dpi (Figures 1I and 1J). While the fraction of naive and early activated cells was somewhat greater at 7 dpi in all organs, the most marked difference was the distribution among cell types in the three organs studied. The mln was characterized by strong GC activity with a smaller proportion of HA-Bmems (3%–6%) and a considerable number of PBs (from 7% on day 7 to 2% on day 28) (Figure 1I). Conversely, few HA^+^ GC B cells were detected in the lungs (~10%), but a remarkably high number of HA-Bmems that rose from 10% on day 7 to ~30% of HA^+^ cells on days 14 and 28. PBs were constant between 1% and 2%. HA^+^ B cells in the spleen exhibited strong GC activity and a relatively constant proportion of HA-Bmems (8%–13%) and PBs (0.5%–1%). Interestingly, PB proportion was the highest in mlns on day 7 but lowest in other organs. This, linked to the mutation analysis (Figure 6) showing nearly germline BCRs in PBs, indicates an early and selective expansion of PBs in the mln. Coordinately, we detected a burst of HA-Bmems in mlns on day 7.

Overall, these data demonstrate early expansion and differentiation of activated HA-specific B cells into PBs and Bmems in the mln and a gradual increase of HA-Bmems in the lungs, which seemed to persist during the response.

### Identification of Bmem precursors using scRNA-seq

**Figure 2:**
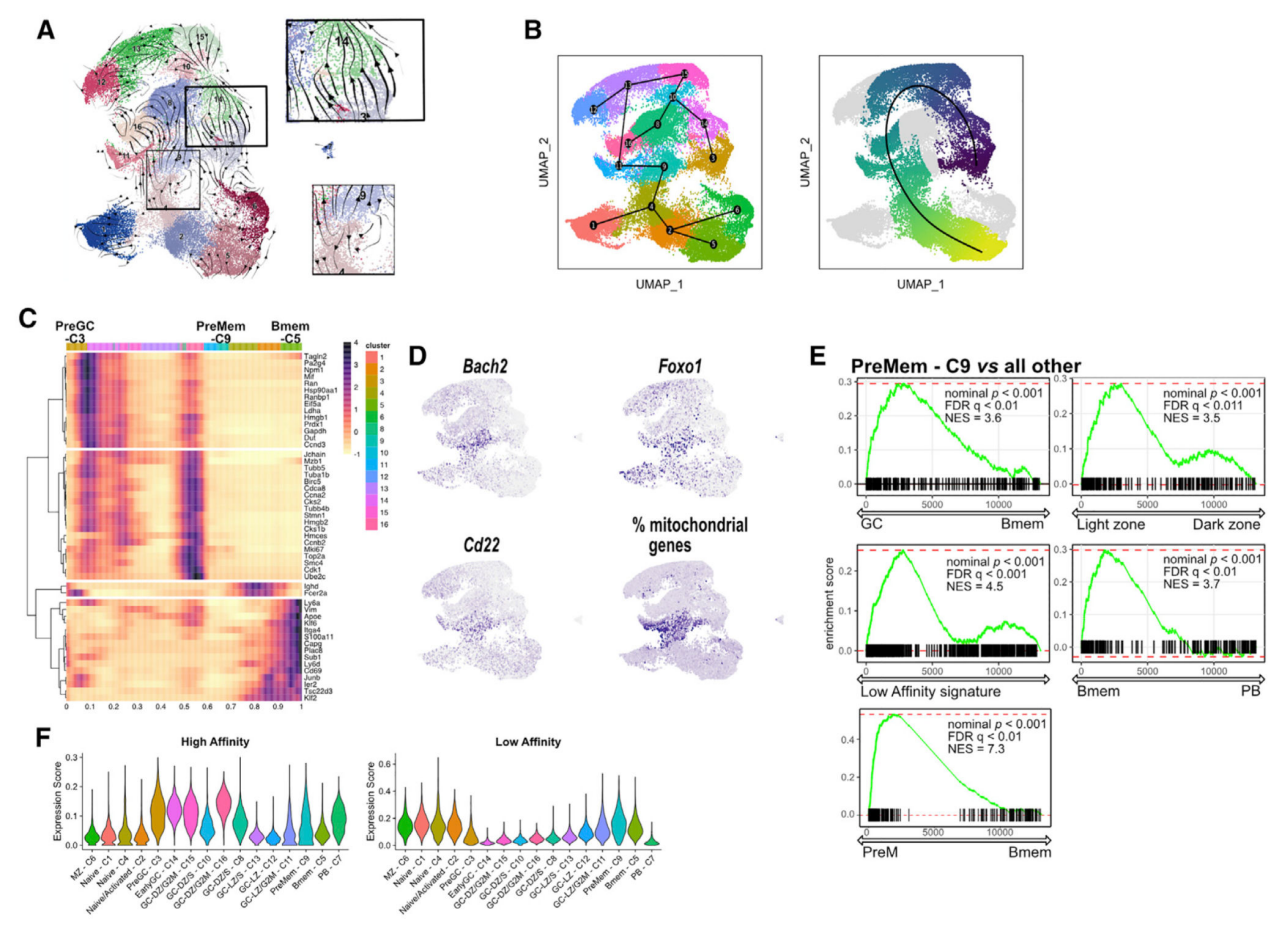
RNA velocity and trajectory analysis identify cluster 9 as memory B cell precursors (A) RNA velocity, as determined by scVelo, projected onto a UMAP. Arrowheads determine predicted direction of the cell movement, and arrow size determines strength of predicted directionality. In the squares are highlighted cells moving from PreGC-C3 to earlyGC-C14 (top) and cells moving from PreMem-C9 (bottom). (B) Trajectory inference by Slingshot projected onto a UMAP with PreGC-C3 selected as the starting cluster. On the right, the same graph is shown with pseudotime coloring. Cluster PB-C7 was excluded because it was clearly disconnected from the others. (C) List of differentially expressed genes over trajectory-based pseudotime. Colors on top indicate clusters. (D) UMAP plot of GC clusters showing average expression of selected genes. (E) Enrichment score from GSEA comparing PreMem-C9 to all others for genes involved in the GC program; the LZ program; the memory cell program; a low-affinity signature, as described by Shinnakasu et al. (2016); and the PreM cluster, as defined by Laidlaw et al. (2020). (F) Violin plot showing high- and low-affinity gene expression scores by UMAP clusters, as defined by Shinnakasu et al. (2016). Data are presented as medians and interquartile ranges

The dynamics of GC B cells have not previously been studied at single-cell resolution, on different days, and in different organs after acute viral infection and, further, the identity of Bmem precursors in the GCs is somewhat controversial (Laidlaw et al., 2017, 2020; Shinnakasu et al., 2016; Suan et al., 2017a). To address those issues and decipher the pattern of Bmem differentiation at the single-cell level, we performed trajectory analysis using Slingshot (Street et al., 2018) and RNA velocity analysis with scVelo (Bergen et al., 2019). RNA velocity analysis suggests that preGC C3s differentiate to earlyGC C14s and subsequently enter GCs (Figure 2A), in accordance with GSEA (Figure 1G). Further, that analysis suggested that PreMem C9s could potentially have some backflow into Bmems (C5). For trajectory analysis with Slingshot, we removed cluster PB C7 because that cluster was clearly disconnected from all others. The trajectory analysis, with preset start at preGC C3, showed a major trajectory going from LZ to DZ to LZ again, with cells exiting from preMem C9 and differentiating to HA-Bmem C5s (Figure 2B). Changes of gene expression along Slingshot pseudotime showed a marked switch in transcriptional programming at preMem C9 (Figure 2C). The preMem C9 cluster expressed several GC marker genes *(Mki67, Aicda,* and *Bcl6)* as well as genes such as *Foxo1, Bach2,* and *Cd22* and have high mitochondrial content (Figure 2D). All these genes and features have been implicated in Bmem development (Chappell et al., 2017; Jang et al., 2015; Shinnakasu et al., 2016). Altogether, this cell population closely resembles the Bmem-precursor cell population previously identified by Shinnakasu et al. (2016). Further, our analysis suggested that cells exiting the GC could undergo intermediate states that can be captured in C4 and C2, hence, partially explaining the mixture of cell states identified in these clusters.

To further confirm that preMem C9 was a Bmem-precursor cluster, we ran a series of GSEA analyses. First, we defined high- and low-affinity scores based on average expression of genes corresponding to high- and low-affinity cells in Shinnakasu et al. (2016). GSEA analysis identified preMem C9 as a LZ GC B cell population with a low-affinity signature as well as being more similar to Bmems than to PBs (Figure 2E). In fact, when assigning low- and high-affinity scores to clusters, preMem C9 had the highest “low-affinity score” among all GC-like clusters, whereas other GC clusters were enriched for the “high-affinity” signature (Figure 2F). Furthermore, preMem C9 cells also showed expression of genes identified by Laidlaw et al. (2020) as expressed in pre-memory cells (Figure 2E), despite differences that exists between our models. Thus, cluster preMem C9 likely represents a Bmem precursor population in the GC, characterized by high *Mki67, Bcl6, Cd22, Bach2,* and *Foxo1* expression and high mitochondrial content.

### Bmems in the lungs have a distinct transcriptional profile, independent of isotype

**Figure 3:**
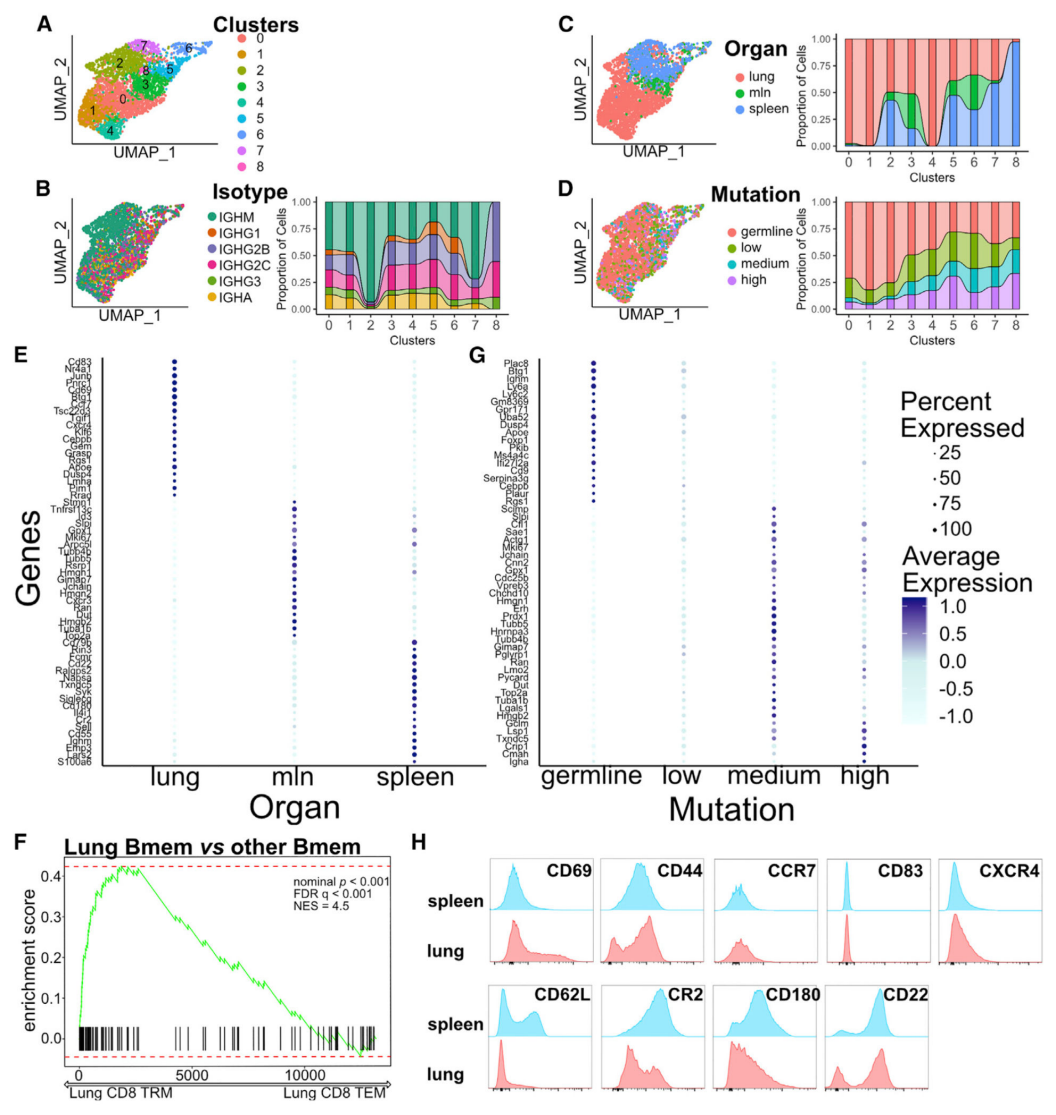
Memory B cells in the lungs have a distinct transcriptional programming compared with that of spleen and mln (A) UMAP plot of unbiased clustering of HA-specific memory B cells (C5 in Figure 1C), combining all organs and dpi. (B) UMAP plot of unbiased clustering of HA-specific memory B cells, as in (A), colored by the BCR isotype. On the right, an alluvial plot shows the proportion of cells with a defined isotype per cluster. (C) UMAP plot of unbiased clustering of HA-specific memory B cells, as in (A), colored by organ. On the right, an alluvial plot shows the proportion of cells belonging to a specific organ per cluster. (D) UMAP plot of unbiased clustering of HA-specific memory B cells, as in (A), colored by BCR mutation rate. Germline (not mutated), low (up to 1% nucleotide mutation), medium (up to 2%), and high (more than 2% mutation). On the right, an alluvial plot shows the proportion of cells with a defined mutation rate per cluster. (E) Mean expression of the top-20 marker genes for each organ for Bmems. Color intensity denotes average expression, whereas dot size shows the percentage of cells expressing the gene. (F) Enrichment score from GSEA comparing lung Bmems to all others for genes expressed by CD8 TRM. (G) Mean expression of the top-20 marker genes for cells divided by mutation rate for Bmems. Color intensity denotes average expression, whereas dot size shows the percentage of cells expressing the gene. (H) Flow cytometry histograms showing expression of the indicated genes by memory B cells (Dump^−^ B220^+^ CD38^+^ IgD^−^ IgM^−^) in lungs and spleen. Representative results of three biological replicates with three mice each.

Next, we hypothesized that Bmem transcriptional profile may be distinct when present in different organs. To address that question, we performed unsupervised analysis of the HA-Bmem population (C5) only. This revealed eight subclusters, with the main determinant of separation being lung versus non-lung localization (Figures 3A–3D). Clusters 0, 1, and 4 were almost exclusively made from lung HA-Bmems, with most other clusters encompassing cells from different organs (Figure 3C). Both GC-dependent and -independent cells, represented by mutated and unmutated BCR sequences and cells of different isotypes, with the exception of cluster 2, which was dominated by IgM, were present in all clusters (Figures 3B and 3D). Although the number and proportion of IgA HA-Bmems in the lung was higher compared with that of spleen (Figure S2A), the B cell heavy-chain class was only a partial determinant in the cell segregation.

Several genes strongly contributed to differential clustering between lung and spleen/mln clustering (Figure 3E). Interestingly, among the most differentially upregulated genes in the lungs were *Cd69,* an adhesion molecule linked to tissue residency of immune cells, together with *Cd83, Ahr, Ccr7, Cxcr4,* and *Cd44.* Conversely spleen and mln had significantly higher *Sell* expression (encoding CD62L), together with Cd22, Cr2, *Bcl2* and *Cd55* (Figure 3E). GSEA analysis on CD8 tissue resident memory signatures revealed striking similarity of lung HA-Bmem to CD8 tissue-resident memory (TRM), as opposed to spleen and mln HA-Bmem (Figure 3F) (Mackay et al., 2013).

Validating the observed differences, PB and GC transcriptional profiles appeared largely similar between organs (Figures S2D–S2K). The only detected difference was between IgA PB and others, as previously reported (Figure S2E) (Neu et al., 2019; Price et al., 2019). Thanks to our approach, we detected transcriptional differences in HA-Bmem between germline (likely GC-independent) and mutated (GC-dependent) cells, suggesting long term functional differences, depending on cell origin (Figures 3G and S2B). Germline HA-Bmem expressed higher levels of *Btg1, Foxp1, Plac8,* and other genes that control cell proliferation and differentiation. On the other hand, highly mutated HA-Bmems (GC dependent) expressed higher levels of *Jchain* (IgA specific), *Slpi, Txndc5, Cmah,* and others associated with programming toward PB differentiation. Likewise, PB also transcriptionally segregated based on mutation rate (Figure S2L).

To validate the transcriptional differences detected in HA-Bmems, we infected mice and analyzed lung and spleen B cells at 14 dpi by flow cytometry. This revealed upregulation of CD69 and CD44 in lung Bmems consistent with scRNA-seq data. CD83 and CXCR4, even if upregulated at the mRNA level, were not detected on the Bmem surface, as expected given their role in GC. Splenic Bmems had higher CD62L, CR2, and CD22 expression levels consistent with scRNA-seq data (Figure 3H).

Collectively, these data demonstrate a distinct transcriptional profile for Bmems in the lung, with hallmarks of activation and tissue residency. The low number of HA^+^ GC B cells in the lung suggest that most lung Bmems are either GC independent or derived from GCs in other organs, which acquire new transcriptional signatures once they take residence in the lungs.

### Vh gene usage is mainly organ specific but is shared among cell types

**Figure 4:**
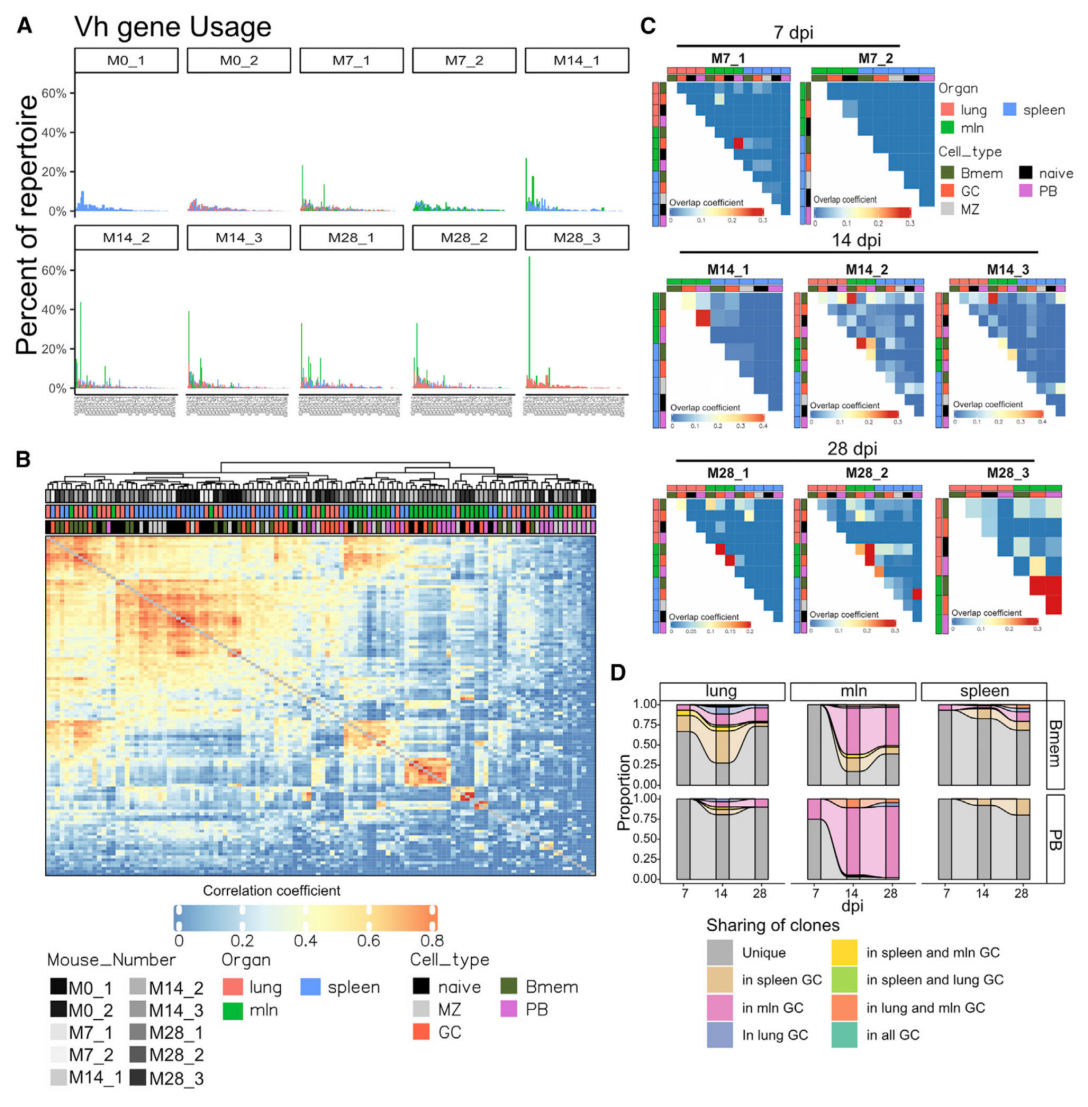
Memory cells broadly disseminate in several organs (A) Percentage of cells using a specific Vh gene for each mouse, divided by organ. (B) Hierarchical clustering of Pearson’s correlation of the V gene repertoire. Each tile represents the correlation of the V gene repertoire. Color intensity indicates correlation strength. See Figure S4 for p values. (C) Overlap between B cell clones in different organ and cell types, divided by mouse. Each tile represents the overlap coefficient of clones. Color intensity indicates overlap strength. (D) Alluvial plots showing clonal origin of Bmems and PBs based on CDR3 sequence, with germline (GC independent) cells excluded from analysis. Top row shows Bmems, and bottom row shows PBs, divided by organ at each dpi. Grey bar indicates that the clones were not found in any GCs, whereas the color indicates that clonal relatives were found in GCs.

Whether there is a bias in Vh gene usage and clonotype for cells differentiating along the PB versus Bmem axes remains a matter of debate. To investigate that question, we assigned V gene usage according to International ImMunoGeneTics (IMGT) standards (Giudicelli et al., 2011). As expected, cells from the unselected naive repertoire were composed of many Vh genes, whereas we could start observing selection already by day 7 (Figure 4A). At 7 dpi, Vh1-63-expressing B cells (Cb site specific) dominated the PB and GC response on day 7 (Figure S3A), as previously reported (Angeletti et al., 2017; Kavaler et al., 1990; Rothaeusler and Baumgarth, 2010), producing germline or near-germline Abs (Figure S3B). Extending previous findings, we also found an increased proportion of these B cells within the Bmem compartment in unmutated form, indicating that these B cells, with high-avidity germline BCRs not only dominated the extrafollicular PB response but also differentiated into IgM and switched HA-Bmem (Figure S3C). On day 14, for all individual mice, Vh gene usage became far more polarized, indicating vigorous clonal selection. Critically, by day 28, one Vh family dominated in each mouse, ranging from 35% to 62% of the response (Figure 4A).

When comparing cell clusters at different time points, our data show some Vh genes undergo selection as early as 7 dpi in GCs and that dominant genes also appear among PBs at 14 dpi (Figure S3D). Finally, we detected some skewed Vh usage in the HA-Bmem population at 28 dpi, consistent with prolonged GC selection (Figure S3D). Selection of D and Jh genes was mainly cell type specific (Figures S3E and S3F). V-gene usage was private to individual mice, except for three genes (Vh14-2, Vh1-81, and Vh1-63) (Figure S3G).

We then analyzed the Vh-gene usage overlap among different mice, organs, and cell types, as defined by uniform manifold approximation and projection (UMAP) clusters, using a Pearson’s correlation matrix (Figures 4B and S3H) (Greiff et al., 2017a). That analysis provided information on overall Vh-gene usage, selection, and clonal expansion and revealed that (1) the V-gene repertoire is mostly clustered by high similarity between lung and spleen B cells, (2) numerous Vh genes can be used to generate HA-binding Abs, (3) most of these genes can be selected into GC and HA-Bmem compartments, whereas (4) selection in to PB compartment is limited to much fewer Vh, which are often also present within the GC and HA-Bmem compartments.

Analyzing clonal overlap based on both heavy and light chains (Figure S3I), revealed that clustering is dominated by differences among mice, extending many prior findings (Greiff et al., 2015, 2017a, 2017b; Miho et al., 2019) that, even in mice with nearly identical genetic backgrounds, most B cells generate selected repertoires that emerge stochastically.

### Bmem cells disseminate in several organs

The high number of Bmems in the lungs, together with the low GC activity, made us hypothesize that most Bmems found there would originate from GCs in other organs. To test whether that was the case, we studied CDR3 clonal overlap within individual mice, organs, and cell types. We found that clonal overlap was mostly organ specific. However, there were notable exceptions, and in several mice, the HA-Bmem populations in the lungs overlapped with GC and HA-Bmem populations in mlns. Interestingly, PBs in mlns were strongly correlated with mln GC in most mice, but the same was not always true for PBs in spleen and lungs (Figures 4C and S3J).

We further investigated clonal sharing between PBs, HA-Bmems, and GCs in different organs. We only considered mutated, likely GC-dependent, clones. By day 14, we could assign almost all PBs in mlns as having a GC origin by clonal relationship (Figure 4D). This was not true for most cells in the lung and spleen because of, at least in part, a low degree of clonal expansion and limited sampling. However, it should be noted that, at least for the spleen and mln, overall diversity for each cluster was similar (Figures S4A and S4B). For HA-Bmems in the spleen, we identified clonal relatives for only ~20% of the cells. This could be due to high diversity and smaller clonal families that make sampling limiting. We could, however, track as many as 75% of HA-Bmems in the lung and mln on day 14 (Figure 1H), despite their high diversity (Figures S4C and S4D). Surprisingly, we observed sharing of GC-derived HA-Bmems, with spleen GCs being a source of HA-Bmems in mlns and lungs and mln-derived HA-Bmems present in the lungs and spleen. These data are consistent with a high degree of dissemination of GC-derived HA-Bmems among organs.

### Clonal expansion is organ specific, and highly expanded clones seed both PB and Bmem compartments

**Figure 5:**
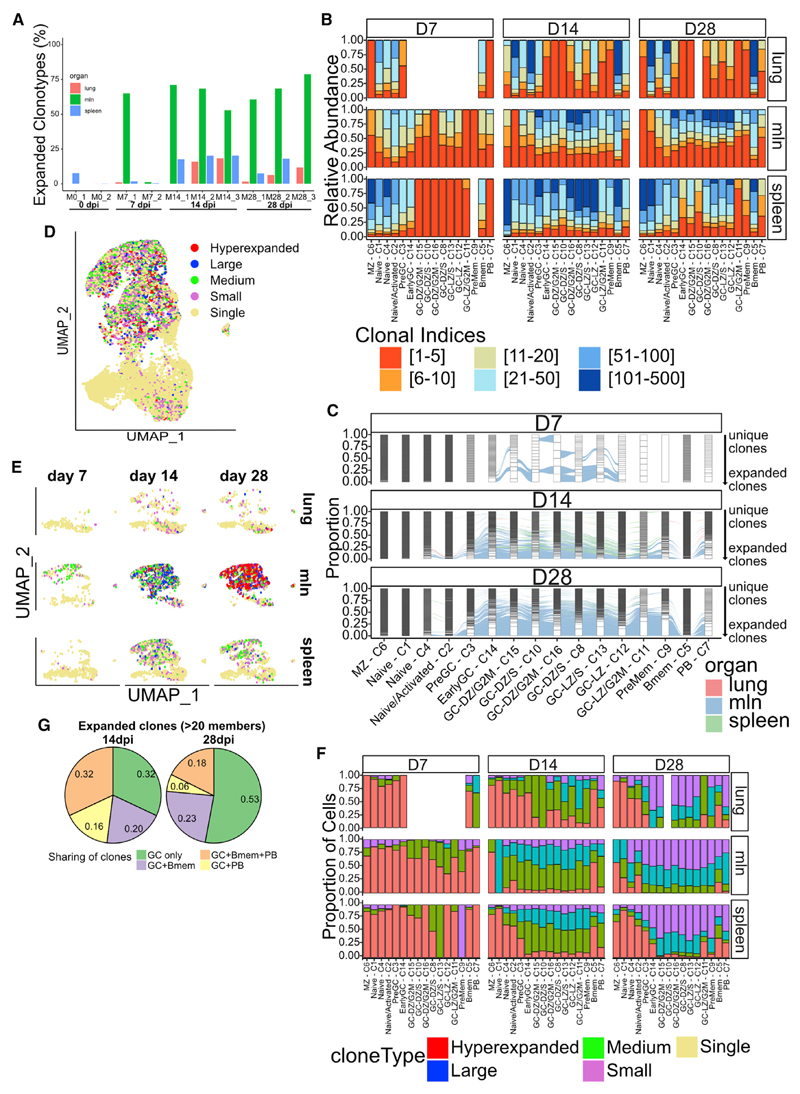
Clonal expansion in GC is organ specific (A) Graph showing the percentage of expanded clonotypes for each mouse, divided by organ. (B) Graph showing clonal expansion for each cluster, divided by dpi and organ. Clones are ordered by their abundance for each cluster, dpi, and organ, and color indicates the repertoire space occupied by the top-X clones as shown in figure legend. (C) Alluvial plots showing clonal sharing between clusters and organs at different dpi. Each cluster is divided in several bars, representing individual clones, and the height of each represents the proportion of the clusters occupied by that clone. Connecting lines indicate the sharing of clones, with colors indicating the organ. (D) UMAP plot of infected cells for all organs and dpi, colored by clonal expansion status. Clones were defined as single (1 cell), small (between 1 and 5 cells), medium (between 6 and 20 cells), large (between 21 and 100 cells), and hyperexpanded (more than 101 cells) clones. (E) UMAP plot of infected cells divided by organ and dpi, colored by clonal expansion status. (F) Graphs showing proportion of cells for each clonal-expansion status for each cluster, divided by dpi and organ. (G) Pie charts showing the distribution of expanded clones (more than 20 cells sequenced) in different clusters. Numbers in the chart indicate frequency.

Having found that clones were shared among several cell types, we asked whether there would be a bias in selection depending on the clonal expansion status. Up to 75% of mln antigen-specific B cells belonged to expanded clonotypes. By contrast, at most 20% of B cells present in the lung/spleen were expanded (Figure 5A). Consistently, the top-50 expanded clones represented more than 50% of the repertoire in mlns, but only 15%–30% in lungs and spleen (Figure S5A). Expectedly, we detected no sign of clonal expansion in naive mice. Analyzing clonal expansion in different clusters, organs, and dpi revealed lower expansion in splenic GC B cells compared with that of mlns (Figure 5B).

HA-Bmem had the most BCR-diverse compartment and had more unique clonotypes (Figure S4). Conversely, PBs, which started with a few highly expanded clonotypes on day 7, became more diverse by day 14 and narrowed again over the next 2 weeks. We generated alluvial graphs to assess the extent to which highly expanded clonotypes are shared among clusters or preferentially expanded in certain clusters over time (Figure 5C). On day 7, only a few clonotypes in the GC clusters were shared among multiple clusters, regardless of the state of clonal expansion. By day 14, 30%–50% of the highly expanded clones were present in all GC clusters, and most of the highly expanded clones also populated HA-Bmem and PB subsets. Clonal sharing among GC, HA-Bmem, and PB was independent of clonal size. On day 28, more than 50% of the BCR sequences were shared among GC clusters, in particular, among highly expanded clones. Consistent with the state of clonal expansion, about 50% of the PBs were derived from highly expanded clones. As in previous dpi, HA-Bmems originated not only from highly expanded GC families but also from smaller clones (Figure 5C). When examining individual mice, the day-14 mice M14_2 and M14_3 stood out, showing that most of the clonal sharing was among splenic GCs and not mln GCs, whereas M28_3 at day 28 had more than 75% clonally expanded cells shared among clusters (Figure S5B).

We visualized clonal expansion by arbitrarily dividing clones into five categories: single (1 cell), small (between 1 and 5 cells), medium (between 6 and 20 cells), large (between 21 and 100 cells), and hyperexpanded (more than 101 cells) clones and rendered them on the UMAP plot (Figure 5D). This clearly demonstrated that most of the expanded cell clones were in the GCs but were also in PB and HA-Bmem clusters. As expected, cells in naive and MZ clusters mostly belonged to single clones. Interestingly, clones in PreGC-C3, which are likely made up of cells just entering the GCs, were mostly unique, consistent with our hypothesis.

Splitting the UMAP according to organ and day (Figures 5E, 3F, and S5C) revealed that GC clonal expansion is organ dependent. In mlns, medium expanded clones are already present on day 7 and are large and hyperexpanded clones by day 14. Conversely, splenic GCs had few medium-sized clones by day 14 and maintained an essentially unmutated expansion profile on day 28. Likewise, lung GC clones were mostly single or small with occasional medium-sized clones appearing. These findings are quite surprising because they suggest organ-dependent regulation of GC clonal expansion. Notably, the number of analyzed GC cells in mlns and spleens on day 14 is nearly identical (n = 2,125 versus 1,828). Nevertheless, to verify that sampling differences didn’t affect our day 28 observation, we randomly downsampled mlns on day 28 to the same number of GC cells as those of spleen and reassigned the expansion status of clonotypes (Figure S5D). We still detected most GC B cells to be either large or hyperexpanded, in stark contrast with spleen GCs, validating our conclusion.

Focusing on highly expanded clones (more than 20 cells), we could define four patterns: clonotypes present in GCs only, GCs and PBs, GCs and HA-Bmems, and GCs, HA-Bmems, and PBs. Remarkably, the proportion of highly expanded clones found only in the GCs increased from 33% to about 50% from day 14 to day 28. Conversely, the fraction of GC clones shared only with PBs decreased, whereas the fraction of HA-Bmems derived from GCs stayed constant by 28 dpi (Figure 5G). Together with the overall clonal-expansion status, this observation indicates that a constant number of GC-derived HA-Bmems are generated as the immune response progresses, whereas the PB diversity decreases as PB clones expand.

### GC-derived PBs and Bmems have similar mutation rates and avidity for antigens

According to previous studies, antigen avidity has a clear role in determining B cell fate. To investigate that, we combined mutation analysis with clonal-expansion data and monoclonal antibody (mAb) expression of HA-specific B cells.

**Figure 6:**
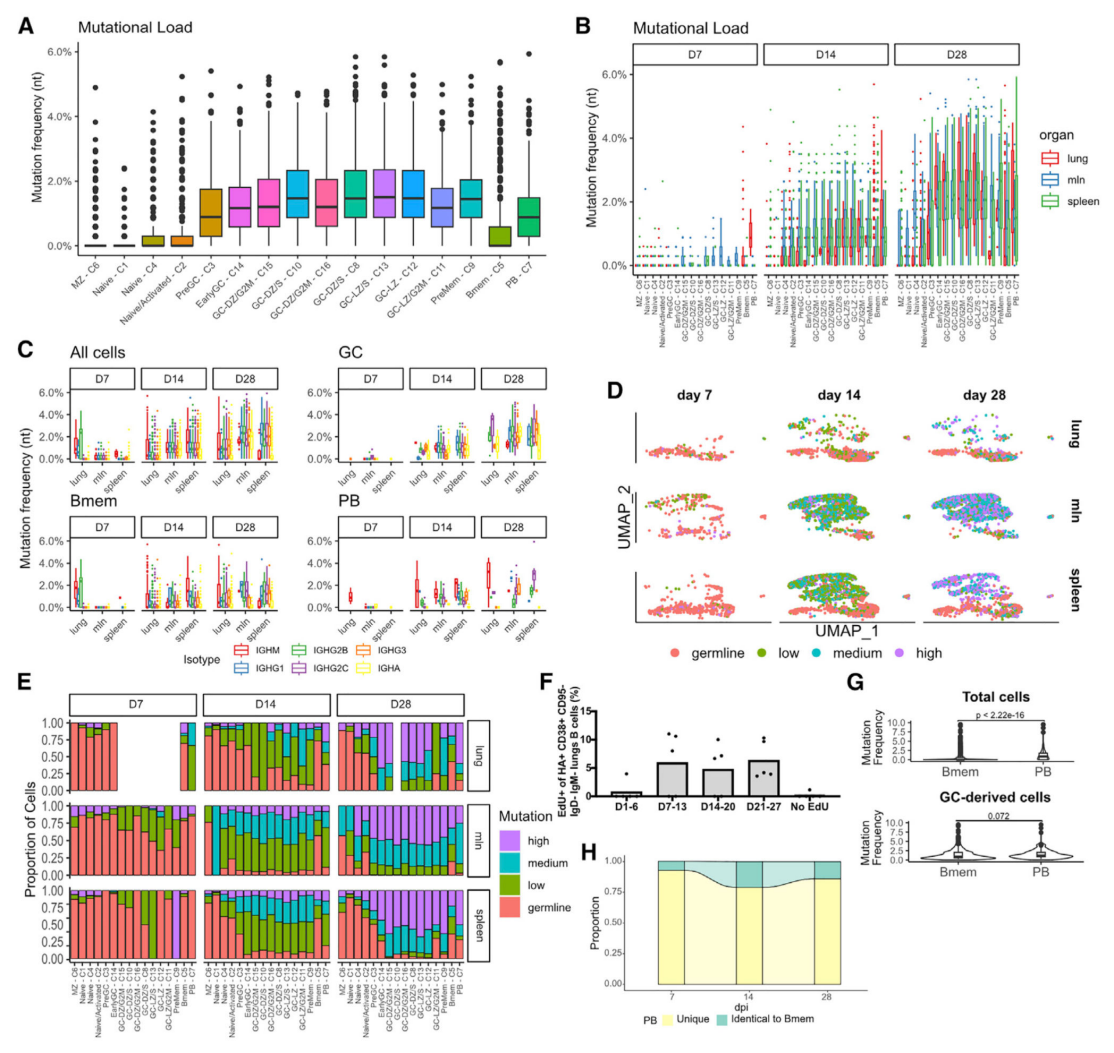
Sustained generation of highly mutated Bmems (A) Graph showing Vh gene mutation frequency divided by UMAP clusters as in Figure 1C. Data are presented as the median and interquartile range. (B) Graphs showing Vh gene mutation frequency for each cluster, divided by dpi and organ. Data are presented as median and interquartile range. (C) Graphs showing Vh gene mutation frequency for each organ, divided by dpi and isotype. Two-way ANOVA with Tukey’s post test: for “All cells” at day 14, each isotype versus IgM, p < 0.0001; IgA versus IgG2b, p < 0.05; IgA versus IgG2c, p < 0.001; IgA versus IgG3, p < 0.01; IgG2c versus IgG1, p < 0.001. For “All cells” at day 28, each isotype versus IgM, p < 0.0001; IgA versus IgG1, p < 0.01; IgA versus IgG2b, p < 0.05; IgG2b versus IgG2c, p < 0.0001; IgG2c versus IgG3, p < 0.01; IgG2c versus IgG1, p < 0.0001; other comparisons ns. For “GC” at day 28, IgG1 versus IgM, p < 0.001; IgG2b versus IgM, p < 0.0001; IgG3 versus IgM, p < 0.01; IgG2c versus IgG1, p < 0.0001; IgG2c versus IgG2b, p < 0.0001; IgG2c versus IgG3, p < 0.0001. For “Bmem” at day 14, IgA versus IgM, p < 0.0001; IgA versus IgG2b, p < 0.0001; IgA versus IgG2c, p < 0.0001; IgA versus IgG3, p < 0.001. For “PB” at day 28, IgA versus IgM, p < 0.0001; IgA versus IgG2b, p < 0.0001; IgA versus IgG2c, p < 0.0001; IgG1 versus IgM. p < 0.01; IgG2b versus IgM, p < 0.01. All other comparisons are non-significant. Data are presented as medians and interquartile ranges. (D) UMAP plots of infected cells divided by organ and dpi, colored by mutation rate. Germline, not mutated; low, up to 1% nucleotide mutation; medium, up to 2%; and high, more than 2% mutation. (E) Graph showing proportion of cells for each mutation rate for each cluster, divided by dpi and organ. (F) Mice were infected with PR8 and injected with EdU at the indicated time windows. At day 35, mice were sacrificed, and lungs were subjected to flow cytometry. Shown is the frequency of EdU^+^ cells among the HA^+^ switched-memory-cell population. The experiment was performed once with n = 5 per group. Data are presented as means. (G) Violin plots comparing mutation frequency of total and GC-derived Bmems versus PBs. Statistical differences were tested using Student’s t test. Data are presented as medians and interquartile ranges. (H) Alluvial plot showing the proportion of PBs with a sequence identical to that of a Bmem, divided by dpi.

In line with what would be expected, naive and MZ cells had almost no mutations, whereas GC cells were, overall, the most mutated, followed by PBs and Bmems (Figure 6A). Separating them by dpi and differentiation clusters, we found that cells from day 7 mice had very few mutations, indicating that PBs and Bmems initially derive from the expansion of unmutated cells. The mutation frequency increased at later dpi, with similar trends for all cluster and organs (Figure 6B). When considering the different heavy-chain classes (Figures 6C and S6A), we did not detect major differences between clusters and organs, with two exceptions: IgM cells were generally less mutated than class-switched cells, and IgA cells tended to have a higher mutation rate, starting from day 14. B cell mutation frequencies among mice were comparable (Figure S6B).

To facilitate mutation analysis, we divided the cells into four discrete bins: germline (not mutated), low (up to 1% nucleotide mutation), medium (up to 2%), and high (more than 2% mutation) (Figure 6D). By day 14, most of the GC cells carried BCRs with low to medium mutations, whereas on day 28, they had medium/high mutation rates. Comparing the mutation data with clonal-expansion data (Figure 5F) highlights the different dynamics between mlns and other organs.

Approximately 75% of HA-Bmems remained at germline on day 14 and 50% on day 28 (Figure 6E). Although we cannot determine the timing of their production, the increased proportion of highly mutated HA-Bmems suggest recent GC origin. To confirm that, we performed a labeling experiment, in which we administered 5-ethynyl-2′-deoxyuridine (EdU) intraperitoneally (i.p.) to mice in a 7-day window after infection (days 1–6, days 7–13, days 14–20, and days 21–27). After 35 days, we analyzed lung-switched Bmems and found that HA^+^ Bmems are produced constantly, in agreement with our sequencing data (Figures 6F, S6C, and S6D). More than one half of the unmutated HA-Bmems were of the IgM isotype, but we also detected IgG and IgA (Figure S2C). Unexpectedly, we found that, when excluding non-mutated, likely GC-independent, cells, the overall mutation rates of PB and HA-Bmem BCRs were statistically indistinguishable, with the exception of HA-Bmems in the lungs and spleen on day 28, having lower mutation rates than PBs had in the same organs (Figures 6G and S6E). Similarly, PreMem-C9, identified by trajectory analysis to be HA-Bmem precursors (Figure 2), had mutation rates and clonal expansion profiles that were indistinguishable from all other clusters. In addition, we found that 22% of PBs had BCR sequences identical to that of HA-Bmems. Further, mutation distribution did not correlate with clonal size, with families with only five members already showing members with high mutation rate (Figure S6F). Although an imperfect proxy, higher SHM usually reflects increased Ab binding avidity (Gitlin et al., 2014; Kocks and Rajewsky, 1988; Neu and Wilson, 2016). Indeed, high- and low-avidity signatures correlated with mutation rate (Figure S6G). Finally, we found that up to 25% of PBs had 100% identity with Bmems (Figure 6H).

**Figure 7:**
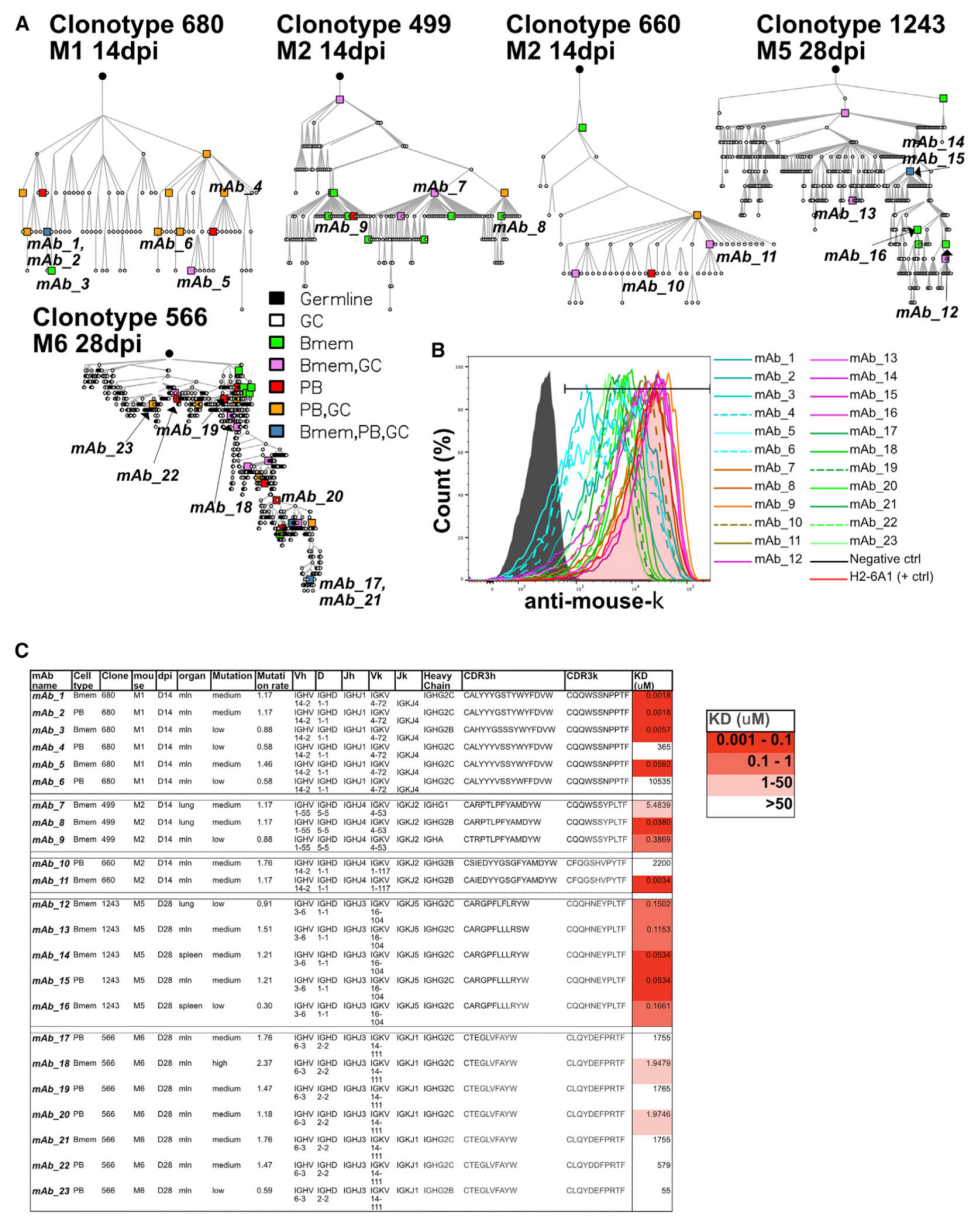
mAbs derived from Bmems and PBs have similar affinity for HAs (A) Clonal trees of five selected clonal families from four mice at different dpi. Color indicates cell types, and each circle or square is a cell that was sequenced in our experiments. Where symbols are missing between junctions, it denotes an inferred member of the clonal family. Expressed mAbs are indicated by name. (B) Madin-Darby canine kidney (MDCK) cells were infected with a PR8-mcherry virus and, at 5 h after infection, were stained with mAbs and detected with anti-mouse *κ*. The histogram shows the binding to viral HA. (C) Characteristics of the expressed mAbs including K_D_ value measured by BLI.

To measure affinity, we expressed mAbs from mutated HA-Bmems and PBs from members of large clonal families. We generated clonal trees for five families (one from M1 and two from M2 at 14 dpi and one each from M5 and M6 at 28 dpi) (Figure 7A). The branching point for differentiation into PBs versus HA-Bmems appears to be random. In more-complex trees, some branches gave rise to both PBs and HA-Bmems.

We expressed 23 representative, switched mAbs (13 from HA-Bmems and 10 from PBs). All the mAbs bound the surface of IAV-infected cells (Figures 7B and S7A), both recombinant and virus-purified HAs, in ELISA (Figures S7B and S7C), albeit with different avidity. We then measured their affinity to HAs by bio-layer interferometry (BLI) (Figure 7C and S7D). Of note, three of the selected mAbs were identical between PBs and HA-Bmems. BLI-affinity measurement showed no pattern of differential affinity between HA-Bmems and PBs. The major determinant of affinity was the clonal family, rather than the cell type or the number of mutations, similar to what was recently reported for HA-Bmem recall after immunization (Mesin et al., 2020). Even in the infected-cell-binding assay, the strength of binding was highly correlated with clonal family. Surprisingly, mAbs from the highly expanded clonotype 566, with more than 700 sequenced cells (>70% of all cells of M28_3) actually exhibited low to extremely low avidity for HAs by BLI. In an extreme example of diverse avidity within a single clonotype, in clone 660, mAb 11 and mAb 10, which differ by two amino acids (with one in the CDR3), exhibit a nearly million-fold difference in K_D_ (2.2 mM versus 3.4 nM). We confirmed the BLI measurements by testing the mAbs by ELISA on HAs and HAs from virus and from PR8 virus treated at pH5 to expose hidden epitopes (Figures S7B–S7D). Interestingly, pH treatment affected mostly mAbs from one clonal family (1243) by decreasing their apparent K_D_, whereas only one mAb had increased K_D_ upon pH treatment (mAb 10). Although the results did not fully recapitulate the BLI measurements, they confirmed that all mAbs bound virus, that there was no difference in apparent avidity between HA-Bmems and PBs within the same family, and that the clonal family was the main determinant in avidity.

## Discussion

B cell responses are the cornerstone of preventing viral infections. A better understanding of how antigen-specific B cell immunity develops after a respiratory viral infection is crucial for designing effective vaccines for influenza viruses, parainfluenza viruses, and, more recently, SARS-CoV-2. How antigen-specific B cells differentiate before GC entry and from the GC to PBs and Bmems remains elusive. Here, by combining sorting of antigen-specific B cells, scRNA-seq and scBCR-seq, we have generated a detailed map of differentiation stages of *de novo* B cells in response to respiratory viral infection. By analyzing events in lungs, draining lymph nodes, and spleen, our data elucidate the complex mechanisms involved in B cell responses to infection.

Most seminal discoveries in B cell biology have, so far, been made in mice immunized with haptens or simple, monovalent protein antigens (e.g., NP, ovalbumin [OVA], HEL, chicken *γ*-globulin [CGG]). Such models do not fully recapitulate the complexity of infectious agents; each of which expresses dozens to hundreds epitopes and, also, idiosyncratically, activates innate immunity, which sculpts the adaptive response. Indeed, recent studies that have used more-complex protein immunogen for immunization (IAV-HA) have challenged established principles of B cell differentiation (Kuraoka et al., 2016; Mesin et al., 2020).

We found that lungs harbor a large population of HA-specific Bmems, much more abundant than expected from the number of iBALT HA-specific GC B cells. This, together with the fact that lung HA-Bmems exhibit a distinct transcriptional profile, lead us to conclude that Bmems generated in other organs emigrate to lungs, as previously speculated, but not formally shown, by Allie et al. (2019). It should be noted that some of the increased expression of activation markers in lungs Bmems could be due to the enzymatic digestion we performed while preparing these tissues. Here, we show that GC-derived Bmems in the lungs can be generated in spleen GCs up to day 14 and in mln GCs up to day 28 and, subsequently, traffic to the lungs where they become tissue resident, by upregulating *Cd69, Cd44,* and *Ahr* and downregulating CD62L (*Sell*), *Cr2, Cd22,* among others. Our findings also highlight the complexity of the Bmem compartment, reflecting a need for specialization and rapid response of Bmems, depending on organ.

Surprisingly, we found different rates of clonal expansion in GCs from mln and spleen, despite similar diversity, even after subsampling to equalize cell numbers. It is possible that clonal bursts (Tas et al., 2016) may be more common in mlns because the total number of GCs is lower or because of increased/persistent antigen levels. Alternatively, clonal expansion could be similar, but splenic GCs may experience increased apoptosis. Whatever the explanation, the net result is the presence of a few hyperexpanded clonal families in mln GCs and many small clonal families in spleen GCs.

Notably, previous studies using NP and HEL immunization models suggested a switch in the output of PBs and Bmems, with early Bmems being unswitched, followed by switched immunoglobulin (swIg), Bmems (between weeks 1 and 2) and, then, on day 21, the generation of PBs (Weisel and Shlomchik, 2017). Except for early IgM Bmems, the response to IAV infection differs from this simplified model, featuring a constant output of PBs and Bmems from GCs, as judged by the mutation rate and EdU-labeling experiment. The strong correlation between clonal size and, importantly, mutational pattern suggests that Bmems are output constantly from GCs.

The origin of Bmem from GCs has been hotly debated. Both inductive and stochastic models for Bmem differentiation have been proposed. Recently, it was proposed that Bmem precursors in the GCs are selected into the memory compartment because of lower affinity as compared with PBs (Shinnakasu et al., 2016; Suan et al., 2017a). This notion is based on NP and HEL immunization using BCR transgenic mice. In both cases, just one amino acid substitution is needed to dramatically improve BCR avidity. To relate this to anti-viral B cell responses, we first examined the prototypical C12 idiotype in the anti-HA response, first described by Kavaler et al (1990), which is specific for the Cb antigenic site of HA. These cells carry a BCR with high germline affinity for HA, rapidly differentiate into extrafollicular plasma cells, and do not participate in secondary responses to flu and, therefore, were assumed not to be forming memory (Kavaler et al., 1991; Rothaeusler and Baumgarth,2010). However, our analysis shows that these cells, of high affinity, are also capable of forming Bmems both through GC-dependent and -independent pathways.

Based on pseudotime analysis and comparison with previously identified gene signatures, we identified PreMem-C9 as Bmem-precursor cells. This cluster was somehow similar to the one identified by Laidlaw et al. (2020); however, in lymphocytic choriomeningitis virus (LCMV) chronic infection, Bmems peak at day 11 (Laidlaw et al., 2017) while they are constantly produced after influenza infection. It is possible that B cells undergo different signaling in chronic, versus acute, infection, and it is unclear how the results are representative of chronic infections in which there is extensive remodeling of lymphoid tissues. Although somatic hypermutation is not a perfect proxy for affinity, it is suggestive that these cells underwent a similar number of selection cycles in the GC. Importantly, we did not sample long-lived plasma cells in the bone marrow; however, after infection, PBs can persist in tissues and mucosa for a long time (Hyland et al., 1994; Jones and Ada, 1987; Khodadadi et al., 2019; Wolf et al., 2011). By expressing a number of mAbs from different clusters of five hyperexpanded clonal families, we found clonality to be the major determinant for affinity differences among B cells, a finding that would have been obviously impossible when using transgenic monoclonal mice. Importantly, soluble mAb expression might not fully recapitulate the complex GC environment in which avidity is also determined by multivalency and BCR density on B cells (Lingwood et al., 2012; Slifka and Amanna, 2019; Tolar and Pierce, 2010). Further, HA used for avidity measurements might not be in the same form presented in GCs on follicular dendritic cells (FDCs), but, nevertheless, the results were reproducible on HAs expressed on the surface of infected cells.

Recently, two studies suggested that even complex antigen Bmems are selected from lower-affinity germline cells (Wong et al., 2020) and that most of them do not bind antigens (Viant et al., 2020). A caveat of our study is that, by using antigen-sorted cells, we might miss B cells of the lowest avidity, which can be activated by multimeric binding *in vivo* (in particular, from the Bmem pool). According to a previous study, these should be approximately 50% of GC cells and 65% of Bmems (Viant et al., 2020) for a tetrameric protein, such as the one used here. Conversely, we might be missing some of the plasma cells that express the lower amount of BCRs on the surface. However, the diversity results from our data suggest that most of the Bmem selection from the GCs might be stochastic (Blink et al., 2005; Good-Jacobson and Shlomchik, 2010; Pélissier et al., 2020; Smith et al., 2000), from both low- and high-affinity precursors, whereas PBs are selected almost exclusively from expanded clones. This is supported by the detection of a high proportion of cells expressing identical BCRs in both HA-Bmem and PB compartments. Our findings are consistent with the previous reports, i.e., we find low-affinity cells only among Bmems, the difference is that, by analyzing a large number of sequences, we are better able to capture a larger fraction of the highly diverse antigen-specific Bmem repertoire. As previously suggested (Baumgarth, 2013), the goal of such a diverse Bmem population would be to sample as much as the HA-reactive repertoire as possible for the host to be prepared for subsequent infection with a variable virus. Our data indicate a possible mechanism behind “original antigenic sin” (Fazekas de St. Groth and Webster, 1966) and highlight current challenges to designing immunogens that are able to recall broadly neutralizing Bmem cells of defined specificity from a highly diverse repertoire.

## STAR⋆Methods

### Key Resources Table

**Table:** 

REAGENT or RESOURCE	SOURCE	IDENTIFIER
Antibodies		
Hamster anti-mouse CD3e BV510	BD Biosciences	563024; RRID:AB_2737959
Rat anti-mouse/human CD45R/B220 PE/Cy7	Biolegend	103222; RRID:AB_313005
Rat anti-mouse/human CD45R/B220 APC/Cy7	Biolegend	103224; RRID:AB_313007
Mouse anti-mouse NK-1.1 BV510	Biolegend	108737; RRID:AB_2562216
Rat anti-mouse CD38 FITC	BD Biosciences	558813; RRID:AB_397126
Rat anti-mouse IgD BUV395	BD Biosciences	564274; RRID:AB_2738723
Rat anti-IgD Pacific Blue	Biolegend	405712; RRID:AB_1937244
Rat Anti-Mouse IgD BV786	BD Biosciences	563618; RRID:AB_2738322
Rat anti-IgM BUV395	BD Biosciences	564025; RRID:AB_2738550
Hamster anti-mouse CD69 BUV737	BD Biosciences	564684; RRID:AB_2738891
Rat anti-mouse CD62L BV711	Biolegend	104445; RRID:AB_2564215
Rat anti-mouse CCR7 BV605	Biolegend	120125; RRID:AB_2715777
Rat anti-mouse CD44 PE/Dazzle 594	Biolegend	103055; RRID:AB_2564043
Rat anti-mouse CD180 BV711	BD Biosciences	740765; RRID:AB_2740428
Mouse anti-mouse CD22.2 BUV737	BD Biosciences	741732; RRID:AB_2871102
Rat anti-mouse CXCR4 PE/Dazzle 594	Biolegend	146513; RRID:AB_2563682
Rat anti-mouse CD83 PE	Biolegend	121507; RRID:AB_572014
Rat anti-mouse CR2/CR1 BV421	Biolegend	123421; RRID:AB_10965544
Rat anti-mouse CD38 BV786	BD Biosciences	740887; RRID:AB_2740536
Hamster anti-mouse CD95 BUV737	BD Biosciences	741763; RRID:AB_2871122
Rat anti-mouse IgG1 PE/CF594	BD Biosciences	562559; RRID:AB_2737654
Rat anti-mouse CD80 BV650	Biolegend	104732; RRID:AB_2686972
Rat anti-mouse CD273 (B7-DC, PD-L2) BV421	Biolegend	107219; RRID:AB_2728127
Rat anti-mouse CD19 Alexa fluor 700	Biolegend	115527; RRID:AB_493734
Rat anti-mouse IgM PE	BD Biosciences	553409; RRID:AB_394845
Streptavidin APC	Invitrogen	S868
HRP Horse Anti-Mouse IgG	Vector Laboratories	PI-2000-1
BV421 Rat Anti-Mouse Ig, κ Light Chain	BD Biosciences	562888; RRID:AB_2737867
Bacterial and virus strains		
TOP10	Invitrogen/ThermoFisher	Cat#C404050
Mouse Influenza A/Puerto Rico/8/34 (PR8) influenza strain grown in 10 day old embryonated chicken eggs	This lab	N/A
Mouse Influenza A/Puerto Rico/8/34 (PR8) influenza strain grown in 10 day old embryonated chicken eggs	(Kosik et al., 2019)	N/A
Chemicals, peptides, and recombinant proteins		
Recombinant antibodies	This paper	N/A
AviTagged recombinant hemagglutinin (PR8)	Mammalian Protein Expression Core Facility (University of Gothenburg)	N/A
Critical commercial assays		
Hanks Balanced Salt Solution (HBSS)	Lonza	10-527F
DMEM, high glucose, GlutaMAX Supplement, pyruvate	ThermoFisher Scientific	31966021
Click-iT Plus EdU Alexa Fluor 488 Flow Cytometry Assay Kit	ThermoFisher Scientific	C10633
Lung Dissociation Kit, mouse	Miltenyi	130-095-927
LIVE/DEAD Fixable Aqua Dead Cell Stain Kit	Invitrogen	L34957
Chromium Single Cell 5′ Library & Gel Bead Kit	10X Genomics	1000006
Chromium Single Cell 5′ Library Construction Kit	10X Genomics	1000020
Chromium Single Cell V(D)J Enrichment Kit, Mouse B Cell	10X Genomics	1000072
Chromium Single Cell A Chip Kit, 16 rxns	10X Genomics	1000009
Chromium i7 Multiplex Kit	10X Genomics	120262
NextSeq 500/550 High Output Kit v2.5 (150 Cycles)	Illumina	20024907
NovaSeq 6000 S1 Reagent Kit v1.5 (100 cycles)	Illumina	20028319
NextSeq 500/550 Mid Output Kit v2.5 (300 Cycles)	Illumina	20024905
MiSeq Reagent Kit v2 (300-cycles)	Illumina	MS-102-2002
HiTrap® Protein G High Performance	Sigma Aldrich	GE17-0404-03
1-step Ultra TMB-ELISA	Thermo Fisher	34029
EasySep Mouse Pan-B Cell Isolation kit	Stem Cell Technologies	19844
Deposited data		
Raw sequencing data files for single-cell RNA sequencing	This paper	ArrayExpress (E-MTAB-9478)
Raw sequencing data files for single-cell VDJ sequencing	This paper	(ArrayExpress) E-MTAB-9491
Experimental models: Cell lines		
Expi293F	ThermoFisher Scientific	Cat#A14527
MDCK	Lab of Jonathan W. Yewdell	N/A
Experimental models: Organisms/strains		
C57BL/6NTac	Taconic Biosciences	B6-F
Oligonucleotides		
Primer for plasmid sequencing: 5′-CTAACAGACTGTTCCTTTCCATG-3′	This paper	N/A
Recombinant DNA		
Mouse IgG1 Heavy chain expression vector	Lab of Jonathan W. Yewdell	N/A
Mouse kappa chain expression vector	Lab of Jonathan W. Yewdell	N/A
Software and algorithms		
Graphpad Prism 9	Graphpad	RRID: SCR_002798
FlowJo version 10	Tree Star	RRID: SCR 008520
Excel	Microsoft	RRID: SCR_016137
Magellan	Tecan	https://lifesciences.tecan.com/software-magellan
R (version 3.6)	The Comprehensive R Archive Network	https://cran.r-project.org/
RStudio (version 1.1.463)	RStudio, Inc.	https://www.rstudio.com/
Seurat (v3.0.1)	Stuart et al., 2019	RRID: SCR_007322
Sauron	This lab	https://github.com/angelettilab/scMouseBcellFlu
Scripts for scRNA seq processing	This lab	https://github.com/angelettilab/scMouseBcellFlu
tradeSeq	(Van den Berge et al., 2019)	RRID: SCR 019238
Velocyto command line tool	(La Manno et al., 2018)	https://velocyto.org/velocyto.py/
scVelo	(Bergen et al., 2019)	RRID: SCR_018168
Immcantation toolbox (v4.0.0)		
TIgGER	(Gadala-Maria et al., 2015)	https://cran.r-project.org/web/packages/tigger/index.html
SHazaM	(Gupta et al., 2015)	https://cran.r-project.org/web/packages/shazam/index.html
Change-O	(Gupta et al., 2015)	https://changeo.readthedocs.io/en/stable/overview.html
scRepertoire	(Borcherding et al., 2020)	https://github.com/ncborcherding/scRepertoire
Ggalluvial	http://corybrunson.github.io/ggalluvial/	https://cran.r-project.org/web/packages/ggalluvial/index.html
pRESTO	Vander Heiden et al., 2014	RRID: SCR_001782
Ggplot2	https://ggplot2.tidyverse.org/	RRID: SCR_014601
Destiny	(Angerer et al., 2016)	https://bioconductor.org/packages/release/bioc/html/destiny.html
Immcantation	(Gupta et al., 2015; Vander Heiden et al., 2014)	https://immcantation.readthedocs.io/en/stable/
Slingshot	(Street et al., 2018)	RRID: SCR_017012
Fgsea	(Korotkevich et al., 2019)	RRID: SCR_020938
Alakazam	(Gupta et al., 2015)	https://cran.rstudio.com/web/packages/alakazam/index.html
iGraph	https://igraph.org/	RRID: SCR_019225
Octet Software Version 10.0	Forté Bio	https://www.sartorius.com/en/products/protein-analysis/octet-systems-software
PHYLIP	https://evolution.genetics.washington.edu/phylip.html	RRID: SCR_006244
Affinity Designer	Affinity	RRID: SCR_016952
10X Cell Ranger package	10X Genomics	https://support.10xgenomics.com

### Resource Availability

#### Lead contact

Further information and requests for resources and reagents should be directed to and will be fulfilled by the lead contact, Davide Angeletti (davide.angeletti@gu.se).

#### Materials availability

The information and requests for resources and reagents should be directed to and will be fulfilled by the lead contact. All plasmids generated in this study are available from the lead contact with a completed Materials Transfer Agreement.

### Experimental Model and Subject Details

#### Mice

All the experiments were conducted according to the protocols (Ethical permit number: 1666/19) approved by regional animal ethics committee in Gothenburg. C57BL/6 mice were purchased from Taconic Biosciences, Denmark. They were housed in the specific pathogen free animal facility of Experimental Biomedicine Unit at the University of Gothenburg. Female mice, which are eight to twelve weeks old, were used in the experiments.

#### Cell lines

MDCK cells were cultured in DMEM (ThermoFisher Scientific) supplemented with 10% heat inactivated FBS (ThermoFisher Scientific) under 5% CO_2_ atmosphere at 37°C. Expi293F cells were cultured in Expi293 Expression Medium (ThermoFisher Scientific) under 8% CO_2_ atmosphere at 37°C in an orbital shaker (125 rpm).

### Method Details

#### Mice infection

Mice were anesthetized with isoflurane and infected through nasal inoculation with 50 TCID_50_ Influenza A/Puerto Rico/8/34 (PR8) (Molecular clone; H1N1) diluted in HBSS containing 0.1% BSA. For EdU labeling experiments, infected mice were injected i.p. daily with 1mg of EdU per mouse.

#### Cell sorting of hemagglutinin-specific B cells

C57BL/6 mice were infected with PR8 H1N1 virus and were euthanized on different days post-infection. Lungs, spleen and mediastinal lymph nodes (mln) were isolated. The same organs from naive mice were used as controls. Spleen and mln were mashed and passed through a 70μm filter to obtain single cell suspension. Lungs were perfused and processed into single cell suspension using the mouse lung dissociation kit (Miltenyi Biotec) according to manufacturer’s instruction. Splenocytes and lung cells were enriched for total B cells using the EasySep Mouse Pan-B Cell Isolation kit (StemCell Technologies) while whole mln cells were used for downstream processing. The cells were incubated for one hour at 4°C with a cocktail of fluorochrome-labeled antibodies consisting of anti-CD3-BV510 (cat. no: 563024, BD), anti-B220-PE-Cy7 (cat. no: 103222, Biolegend) and anti-IgD-Pacific Blue (cat. no: 405712, Biolegend), and 1μg/ml biotinylated recombinant hemagglutinin (rHA) (Whittle et al., 2014) conjugated to streptavidin APC (cat. no: S868, Invitrogen). To exclude dead cells, the cells were washed and stained with LIVE/DEAD Fixable Aqua Dead Cell Stain (cat. no: L34957, Invitrogen) according to manufacturer’s instruction. A maximum of 10,000 live HA-specific mature B cells (CD3^-^B220^+^IgD^-^rHA^+^) were sorted and collected in a BD FACSAria fusion or BD FACSAria III (BD Biosciences) cell sorter and processed immediately.

#### Flow cytometry

All the fluorochrome-labeled antibodies used in flow cytometry were titrated for determining the optimal concentration. Briefly, spleens and lungs were harvested from C57BL/6 mice on day 14 post-PR8 H1N1 infection after euthanization. Spleens were processed into single cell suspension by mashing them and passing through a 70μm filter. Lungs were processed into single cell suspension using the mouse lung dissociation kit (Miltenyi Biotec). The cells were stained with indicated fluorochrome conjugated antibodies. The complete list of antibodies can be found in Key Resources Table. The cells were stained with fluorochrome labeled antibodies for 20 min at 4°C. After washing, the cells are stained with LIVE/DEAD Fixable Aqua Dead Cell Stain (cat. no: L34957, Invitrogen) to exclude dead cells. For EdU experiments, after surface staining the Click reaction was performed using Click-iT Plus EdU Alexa Fluor 488 Flow Cytometry Assay Kit (Thermo Fisher, C10633), according to manufacturer’s instructions. The labeled cells were run and the data was acquired on the BD LSR Fortessa X-20 (BD Biosciences) and was analyzed using Flow-Jo software (Tree Star).

#### Generation and sequencing of single cell gene expression and enriched B cell libraries

Nearly 1500-10,000 sorted HA-specific mature B cells from individual organs were processed into single cells in a chromium controller (10X genomics). During this process, individual cells are embedded in Gel Beads-in-emulsion (GEMs) where all generated cDNA share a common 10X oligonucleotide barcode. After amplification of the cDNA, 5′gene expression library and enriched B cell library, with paired heavy and light chain were generated from cDNA of the same cell using Chromium single cell VDJ reagent kit (V1.1 chemistry, 10X genomics). The 5′gene expression libraries were sequenced in NextSeq or NovaSeq6000 sequencer (Illumina) using NextSeq 500/550 v2.5 sequencing reagent kit (2 × 75 bp) or NovaSeq S1 sequencing reagent kit (2 × 100 bp) (Illumina) respectively. The enriched B cell libraries were sequenced in NextSeq or MiSeq sequencer using NextSeq Mid Output v2.5 sequencing reagent kit (2 × 150 bp) or MiSeq Reagent Kit v2 (2 × 150 bp) (Illumina) respectively.

Lungs from mice M0_1, M7_2 and M14_1 and spleen from M28_3 failed to yield good quality GEMs and libraries and were not sequenced.

#### Single-cell RNA-seq data processing

Single-cell RNA-seq data was processed in R with Sauron (https://github.com/NBISweden/sauron), which primarily utilizes the Seurat (v3.0.1) package (Stuart et al., 2019). This workflow comprises a generalized set of tools and commands to analyze single cell data in a more reproducible and standardized manner, either locally or in a computer cluster. The complete workflow and associated scripts are available on https://github.com/angelettilab/scMouseBcellFlu. A set of instructions on how to use the workflow and completely reproduce the results shown herein are available there.

Raw UMI count matrices generated from the cellranger 10X pipeline were loaded and merged into a single Seurat object. Cells were discarded if they met any one of the following criteria: percentage of mitochondrial counts > 25%; percentage of ribosomal (Rps or Rpl) counts > 25%; number of unique features or total counts was in the bottom or top 0.5% of all cells; number of unique features < 200; Gini or Simpson diversity index < 0.8. Furthermore, mitochondrial genes, non-protein-coding genes, and genes expressed in fewer than 5 cells were discarded, whereas the immunoglobulin genes *Ighd, Ighm, Ighg1, Ighg2c, Ighg2b, Ighg3, Igha,* and *Ighe* were retained in the dataset regardless of their properties.

Gene counts were normalized to the same total counts per cell (1000) and natural log transformed (after the addition of a pseudo-count of 1). The normalized counts in each cell were mean-centered and scaled by their standard deviation, and the following variables were regressed out: number of features, percentage of mitochondrial counts, and the difference between the G2M and S phase scores.

Data integration across cells originating from different samples, time points and tissues were done on regressed scaled counts using the mutual nearest neighbors (MNN) (Haghverdi et al., 2018) on a set of highly variable genes (HVGs) identified within each sample individually and combined. The top 20 nearest neighbors (k) with a final dimensionality of 51 were used. Uniform Manifold Approximation and Projection (UMAP) (McInnes et al., 2018) was applied to the MNN-integrated data to further reduce dimensionality for visualization (2 dimensions) or for unsupervised clustering (10 dimensions).

At this stage, differential expression between clusters, and cell correlation with cell-type specific gene lists were evaluated to identify clusters of non-B cells (such as NK or T cells). Predicted non-B cells were removed from the data, and the entire single-cell RNA-seq processing pipeline was re-run using only the remaining B cells. Finally, hierarchical clustering was performed on the 10-dimensional UMAP embedding using Ward’s method with Euclidean distances to define 16 clusters of B cell subtypes, which were then visualized on the 2-dimensional UMAP embedding.

#### Trajectory inference analysis and RNA velocity

Trajectory inference analysis was performed on a diffusion map embedding (20 diffusion components; DCs) of the MNN-integrated count data using the destiny package (Angerer et al., 2016). The cell differentiation lineages were then predicted from the DCs using the slingshot package (Street et al., 2018). Cluster 3 (cells entering the GC) was specified as the starting point and cluster 5 as the end point (Bmem). Distance along the resulting curve was used to define the position of each cell in pseudotime. Identification of differentially expressed genes was done by fitting a generalized additive model (GAM) to the trajectory curve using the tradeSeq package (Van den Berge et al., 2019), allowing us to detect which genes exhibited expression behavior that was most strongly associated with progression along the defined lineage. Single-cell RNA-seq BAM files were processed using the velocyto command line tool (La Manno et al., 2018) to quantify the amount of unspliced and spliced RNA reads of each gene in each sample.

The scVelo package (Bergen et al., 2019) was used to perform the RNA velocity analysis. The first- and second-order moments for velocity estimation were calculated using the MNN-integrated data as the representation, and the cell velocities were computed using the likelihood-based dynamical model. A velocity graph was calculated based on cosine similarities between cells, and cell velocities were visualized as streamlines overlaid on the 2-dimensional UMAP embedding.

#### BCR sequence data processing

The BCR sequence data was processed using the Immcantation toolbox (v4.0.0) using the IgBLAST and IMGT germline sequence databases, with default parameter values unless otherwise noted. The IgBLAST database was used to assign V(D)J gene annotations to the BCR FASTA files for each sample using the Change-O package (Gupta et al., 2015), resulting in a matrix containing sequence alignment information for each sample for both light and heavy chain sequences.

BCR sequence database files associated with the same individual (mouse) were combined and processed to infer the genotype using the TIgGER package (Gadala-Maria et al., 2015) as well as to correct allele calls based on the inferred genotype. The SHazaM package (Gupta et al., 2015) was used to evaluate sequence similarities based on their Hamming distance and estimate the distance threshold separating clonally related from unrelated sequences. The predicted thresholds ranged from 0.096 to 0.169, where a default value of 0.1 was assumed for cases when the automatic threshold detection failed. Ig sequences were assigned to clones using Change-O, where the distance threshold was set to the corresponding value predicted with SHazaM in the previous step. Germline sequences were generated for each mouse using the genotyped sequences (FASTA files) obtained using TIgGER (Gadala-Maria et al., 2015). BCR mutation frequencies were then estimated using SHazaM. The BCR sequence data, clone assignments, and estimated mutation frequencies were integrated with the single-cell RNA-seq data by aligning and merging the data with the metadata slot in the processed RNA-seq Seurat object.

#### Identification of clones and diversity using scRepertoire

scRepertoire (Borcherding et al., 2020) was used to determine clonal groups based on paired heavy and light chains. This package uses the filtered contig annotation obtained from cell ranger. Clones were assigned only for cells where high quality paired heavy and light chains were sequenced. Clones were assigned based on the CTstrict function per each mouse. The CTstrict function considers clonally related two sequences with identical V gene usage and > 85% normalized hamming distance of the nucleotide sequence. Percent of unique clonotypes were obtained using the quantContig function. Integration with the Seurat object was done using the combineExpression function. Ranking of clones were determined using the clonalProportion function and Shannon and Simpson’s diversity determined using the clonalDiversity function. All functions were run using the exportTable = T function to obtain a table and customarily facet the graph in R using the ggplot package. Sharing of clones between clusters was visualized using the ggalluvial package.

#### Differential gene expression analysis

Differentially expressed genes between different clusters, organs, isotypes or differentially mutated cells were identified using the FindAllMarkers function from Seurat using default settings (Wilcoxon test and Bonferroni *p* value correction). Significant genes with average log fold change > 0.25 and expressed in > 25% of cells in that group were ranked according to fold change and represented in the FeaturePlot.

#### Gene set enrichment analysis (GSEA)

For GSEA analysis, differentially expressed genes for each cluster or organ were calculated using the Wilcoxon rank sum test via the wilcoxauc function of the presto package using default parameters (including Benjamini-Hochberg false discovery rate correction) and filtered on logFC > 1 and *padj* < 0.05. GSEA was run on pre-ranked genes using the fgsea package (Korotkevich et al., 2019).For each enrichment graph we report *p*, *padj* (FDR *q*) and NES (enrichment score normalized to mean enrichment of random samples of the same size) values in the figure.

#### Generation of clonal trees and expression of monoclonal antibodies

Five clonal families were randomly selected among the hyperexpanded, as defined by scRepertoire. Clonal trees were reconstructed using the Alakazam package of Immcantation (Gupta et al., 2015). In brief, clones were made with the function makeChangeOclones and lineages were reconstructed using the dnapars function of the Phylip package via buildPhylipLineage function. Clonal trees were visualized via the iGraph package in R. Random representative clones were selected from each family. Both heavy and light chain sequences were synthesized (Twist Bioscience) and subsequently cloned into a mouse IgG1 expression vector (from the Yewdell laboratory). To confirm the cloning, developed vectors were sequenced by Eurofins Genomics. To express recombinant antibodies plasmids encoding corresponding heavy and light chains were mixed in equal ratio. Transfection of Expi293F cells was carried out by ExpiFectamine 293 Transfection Kit (Thermo Fisher) according to the manufacturer instruction. After four days supernatants were collected and filtered. Purification of the immunoglobulins was carried out by Akta Start System (GE Healthcare) using protein G column. Elution of bound antibodies was done by 0.1 M glycine buffer, pH 2.7. To neutralize the solution coming from the column collecting tubes contained 1 M Tris buffer, pH 9.0. Antibody-containing eluates were concentrated by centrifugation through VivaSpin columns with a 30 kDa cut-off. Estimation of antibody concentration was done by NanoDrop (Thermo Fisher) equipment. Binding of all mAbs was confirmed using ELISA. Briefly, plates were coated overnight with 20HAU of PR8 virus in PBS. Plates were blocked with PBS 2% milk for 1h at RT. mAbs were serially diluted and incubated for 1hr at RT. After washing, plates were incubated with HRP Horse Anti-Mouse IgG Antibody (Vector laboratorie, cat. no: PI-2000-1, dilution 1:1000) 1hr at RT. Plates were developed with 1-step Ultra TMB-ELISA (Thermo Fisher), reaction stopped by 2M H_2_SO_4_ and read at 450nm in an Tecan microplate reader using Magellen software.

#### Bio-layer interferometry

Biolayer Interferometry (BLI)-based assay was set up to measure the affinity of murine mAbs to Pro-haemeagglutinin (HA). In principle, BLI is an optical analytical, label-free, technique and is used to analyze biological interactions using the difference in interference pattern of white light reflected from two surfaces: a layer of immobilized ligand on the biosensor tip and an internal reference (blank) layer.

Kinetic assays were optimized for buffer, pH, temperature conditions, orbital shake speed for affinity analysis. Briefly, 0.5 mg of AviTagged HA diluted in acetate 4.5 (pall fortebio, Sartorius group, Amsterdam, Netherlands) was immobilized onto SAX biosensors (high precision streptavidin sensors: Pall fortebio, Sartorius group, Amsterdam, Netherlands). mAbs were diluted 1:1000 ratio in kinetic buffer (1%BSA in PBS, pH 7.5) Pall fortebio, Sartorius group, Amsterdam, Netherlands). H26A1, H28-E23 and H17-40 mAbs were used as positive controls.

Kinetics of binding interactions of mAbs to the immobilized HA were determined using octet data acquisition software (version 10.0.087) blank experiment with the following experimental steps: wash (PBS buffer, pH 7.5; 60 s), immobilization (HA, 0.5 mg; 300 s), association (analytes, 1:1000 of mAbs; 420 s), dissociation (PBS buffer, pH 7.5; 600 s), regeneration (10mM glycine, Sigma-Aldrich, Stockholm, Sweden; 60 s), wash (PBS buffer, pH 7.5; 60 s). Experiments were carried out at plate shake speed of 1000 rpm and plate temperature of 25 ° C. Reference sensor was immobilized with PBS (pH 7.5, GIBCO, Sweden) and samples were run with same experimental conditions as ligand immobilized sensor. Data was processed using octet data analysis software (version 10.0) and on-rate (ka), off-rate (kd) and affinity (K_D_) were calculated upon reference subtraction.

#### Infected cells binding assay

The assay was carried out as previously described (Angeletti et al., 2019). MDCK cells were infected using PR8-mCherry expressing virus, at MOI = 5 for 5h at 37°C. After incubation, cells were transferred into tubes and stained with mAbs or control mAbs (H26A1 as positive control and unrelated PE-specific mAb as negative) at 25ug/ml for 60 min at 37°C. After washes with PBS/0.1% BSA cells were stained with BV421 anti-mouse Ig light chain (clone 187.1)(BD, cat 562888) for 30 min at 4°C before acquisition via flow cytometry.

### Quantification and Statistical Analysis

For single cells analyses, statistics are described in Method details section. GraphPad Prism was used for data analysis except for single cell analyses. For multiple comparison between groups, One-way ANOVA or two-way ANOVA with Tukey’s multiple comparisons were used. Violin plots and boxplots are represented as median and interquartile range while other statistical data are presented as mean ± SEM p < 0.05 was considered statistically significant. The number of animals used in the experiment are indicated either in the figure legend or figure.

## Supplementary Material

Supplemental Information

## Data Availability

The complete workflow and associated scripts are available on https://github.com/angelettilab/scMouseBcellFlu. A set of instructions on how to use the workflow and completely reproduce the results shown herein are available there. Raw sequencing data files for single-cell RNA sequencing and single-cell VDJ sequencing are available at ArrayExpress: E-MTAB-9478 and E-MTAB-9491.

## Highlights

- Integrated, single-cell RNA- and BCR-seq in three organs after influenza infection
- Switched, mutated memory B cells are continuously produced from germinal centers
- Lung memory B cells originate from lymphoid organs and assume residency phenotype
- Memory B cells are derived from both low- and high-affinity GC precursors

## In brief

Combining single-cell RNA and BCR sequencing, Mathew et al. report that, after influenza infection, memory B cells (Bmems) are stochastically selected from both low- and high-affinity germinal center (GC) precursors. They reveal that lung Bmems acquire tissue-residency signatures and continuously differentiate from the spleen and mediastinal lymph nodes GCs.
