# Supplementary material for: Single-cell BCR and transcriptome analysis after influenza infection reveals spatiotemporal dynamics of antigen-specific B cells
Source: Cell Rep. Author manuscript; Available in PMC 2022 Jun 30. (PMC7612943; doi:10.1016/j.celrep.2021.109286)
Supplement: Supplemental Information [file EMS146188-supplement-Supplemental_Information.zip › 1-s2.0-S2211124721006574-mmc1.pdf]

Cell Reports, Volume *35*

Supplemental information

Single-cell BCR and transcriptome analysis after influenza infection reveals spatiotemporal dynamics of antigen-specific B cells

Nimitha R. Mathew, Jayalal K. Jayanthan, Ilya V. Smirnov, Jonathan L. Robinson, Hannes Axelsson, Sravya S. Nakka, Aikaterini Emmanouilidi, Paulo Czarnewski, William T. Yewdell, Karin Schön, Cristina Lebrero-Fernández, Valentina Bernasconi, William Rodin, Ali M. Harandi, Nils Lycke, Nicholas Borcherding, Jonathan W. Yewdell, Victor Greiff, Mats Bemark, and Davide Angeletti

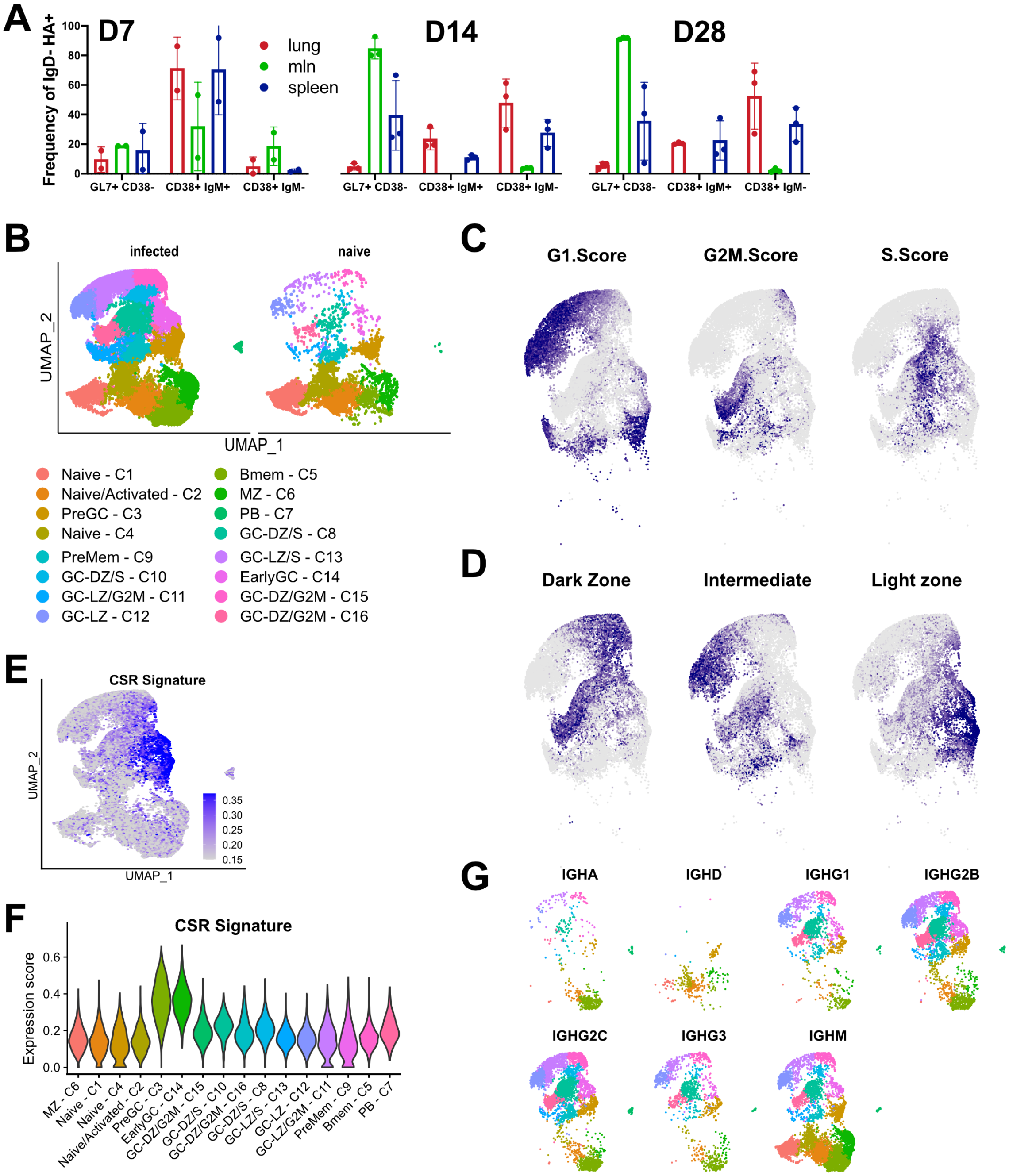

FigS1. Cell cycle, isotype and CSR signatures of B cells, Related to Fig. 1.

A) Frequency (mean±SEM) of sorted cells used for scRNAseq experiments, gated on Live, CD3-, B220+, IgD- HA+.
B) UMAP plot of all mice divided by infection status (infected vs naïve).
C) UMAP plot of GC clusters showing cell cycle phase.
D) UMAP plot of GC clusters showing Dark zone, intermediate and light zone scores according to gene signatures identified by Holmes et al., 2020.
E) UMAP showing class switch recombination (CSR) signature as identified by King et al., 2021.
F) Violin Plot showing the same CSR signature as in E. Data are presented as the median and interquartile range.
G) UMAP plot of infected mice divided by B cell isotype (according to BCR sequencing).

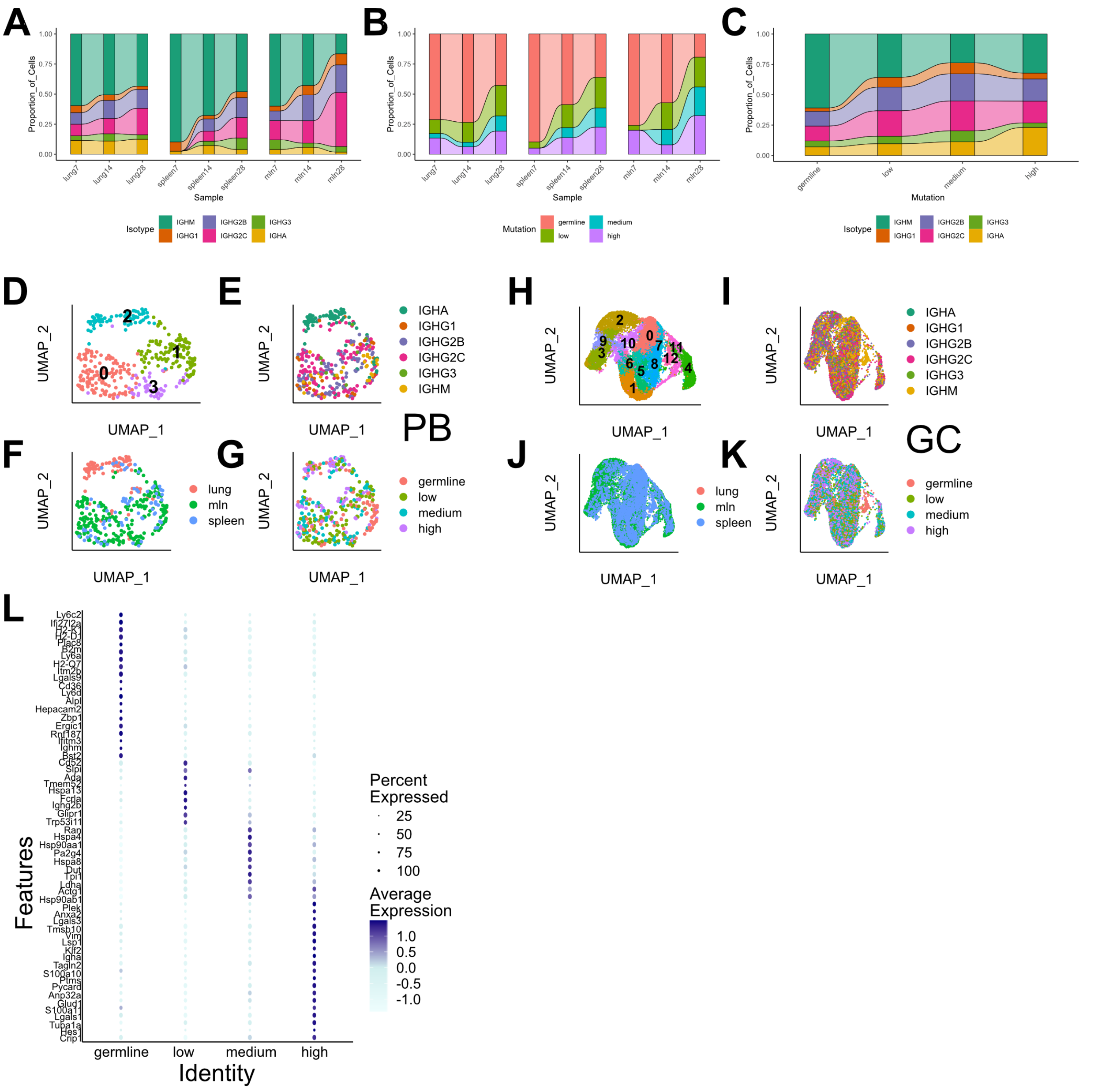

Fig. S2. Characteristics of Bmem, PB and GC cell populations in the lungs, Related to Fig 3.
A) Alluvial plot showing proportion of Bmem with defined isotype per organ at each dpi.
B) Alluvial plot showing proportion of Bmem with defined mutation profile per organ at each dpi.
C) Alluvial plot showing proportion of Bmem with defined isotype per mutation profile.
D) UMAP plot of unbiased clustering of HA-specific Plasmablasts (cluster 7 in Fig 1C), combining all organs and dpi.
E) UMAP plot of unbiased clustering of HA-specific Plasmablasts as in A, colored by BCR isotype.
F) UMAP plot of unbiased clustering of HA-specific Plasmablasts as in A, colored by organ.
G) UMAP plot of unbiased clustering of HA-specific Plasmablasts as in A, colored by BCR mutation rate.
H) UMAP plot of unbiased clustering of HA-specific GC B cells (clusters 3, 9, 10, 11, 12, 13, 14, 15 and 16 in Fig 1C), combining all organs and dpi.
I) UMAP plot of unbiased clustering of HA-specific GC B cells as in E, colored by BCR isotype.
J) UMAP plot of unbiased clustering of HA-specific GC B cells as in E, colored by organ.
K) UMAP plot of unbiased clustering of HA-specific GC B cells as in E, colored by BCR mutation rate.
L) Mean expression of the top 20 marker genes for Plasmablasts divided by mutation rate. Color intensity denotes average expression while dot size the percent of cells expressing the gene.

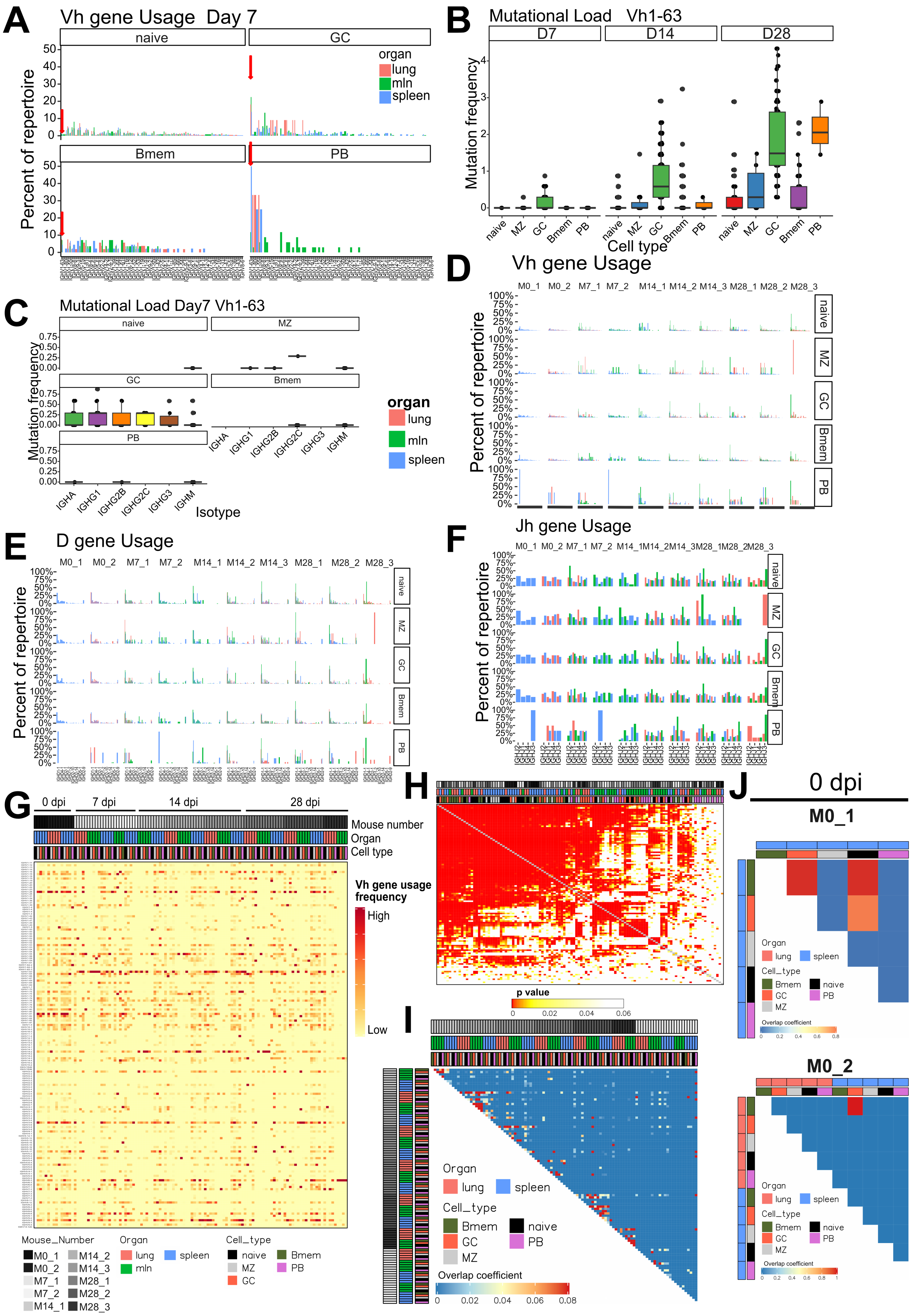
A
Vh gene Usage Day 7
naive
GC
Bmem
PB
organ
lung
mln
spleen
Percent of repertoire
B
Mutational Load Vh1-63
D7
D14
D28
Mutation frequency
Cell type
naive
MZ
GC
Bmem
PB
C
Mutational Load Day7 Vh1-63
naive
MZ
GC
Bmem
PB
Mutation frequency
Isotype
IGHA
IGHG1
IGHG2B
IGHG2C
IGHG3
IGHM
organ
lung
mln
spleen
D
Vh gene Usage
M0_1 M0_2 M7_1 M7_2 M14_1 M14_2 M14_3 M28_1 M28_2 M28_3
Percent of repertoire
E
D gene Usage
F
Jh gene Usage
G
0 dpi
7 dpi
14 dpi
28 dpi
Mouse number
Organ
Cell type
Vh gene usage frequency
High
Low
Mouse_Number
M0_1 M0_2 M7_1 M7_2 M14_1 M14_2 M14_3 M28_1 M28_2 M28_3
Organ
lung
spleen
mln
Cell_type
naive
Bmem
MZ
PB
GC
H
p value
0 0.02 0.04 0.06
I
Organ
lung
spleen
Cell_type
Bmem
naive
GC
PB
MZ
Overlap coefficient
0 0.02 0.04 0.06 0.08
J
0 dpi
M0_1
M0_2
Organ
lung
spleen
Cell_type
Bmem
naive
GC
PB
MZ
Overlap coefficient

Fig S3. V, D and J gene usage in different B cell subpopulation and their clonal relatedness, Related to Fig 4.

A) Percentage of cells using a specific Vh gene for each mouse, divided by organ. Red arrows indicate the Vh gene 1-63.

B) Mutation rate of members of Vh1-63, divided by cell type, at different dpi. Data are presented as the median and interquartile range.

C) Mutation rate of members of Vh1-63 at day 7, divided by cell type and isotype. Data are presented as the median and interquartile range.

D) Bar plot showing Vh gene usage frequency by mouse and celltype, divided by organ.

E) Bar plot showing D gene usage frequency by mouse and celltype, divided by organ.

F) Bar plot showing Jh gene usage frequency by mouse and celltype, divided by organ.

G) Heatmap showing Vh gene usage frequency by mouse, organ and cell types (as identified by UMAP clustering). Each line on the y axis represents a single Vh gene. Frequency was normalized by Vh gene

H) Same matrix as in Fig 4B but depicted here is the p value for each correlation. White tile = non-significant, light yellow $p<0.05$, bright yellow $p<0.01$ and red tiles $p<0.001$.

I) Overlap between B cell clones in different organ and cell types. Each tile represents the overlap coefficient of clones. Color intensity indicates overlap strength.

J) Overlap between B cell clones in different organ and cell types, for the two naïve mice. Each tile represents the overlap coefficient of clones. Color intensity indicates overlap strength.

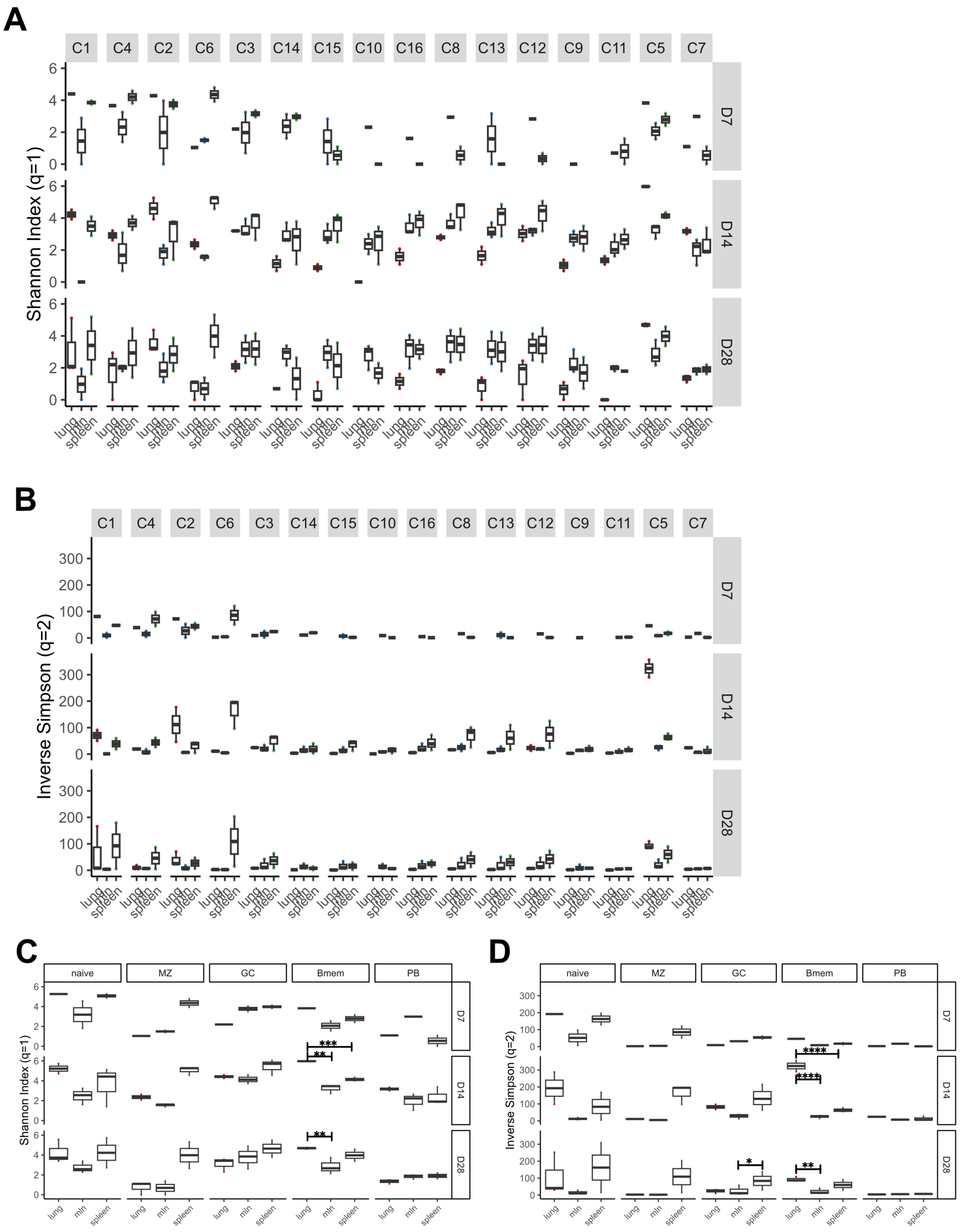

Fig S4. Diversity indices for different B cell subpopulations, Related to Fig 5.

A) Shannon´s diversity index for all cells divided by cluster, dpi and organ. Data are presented as the median and interquartile range.

B) Inverse Simpson diversity index for all cells divided by cluster, dpi and organ. Data are presented as the median and interquartile range.

C) Shannon´s diversity index for all cells divided by cell type, dpi and organ. Data are presented as the median and interquartile range.Two-Way ANOVA followed by Tukey post-hoc test, only significant differences shown. Significance of $P \leq 0.05$ indicated as *, $P \leq 0.01$ as **, $P \leq 0.001$ as ***, $P \leq 0.0001$ as ****.

D) Inverse Simpson diversity index for all cells divided by cell type, dpi and organ. Data are presented as the median and interquartile range.Two-Way ANOVA followed by Tukey post-hoc test, only significant differences shown. Significance of $P \leq 0.05$ indicated as *, $P \leq 0.01$ as **, $P \leq 0.001$ as ***, $P \leq 0.0001$ as ****.

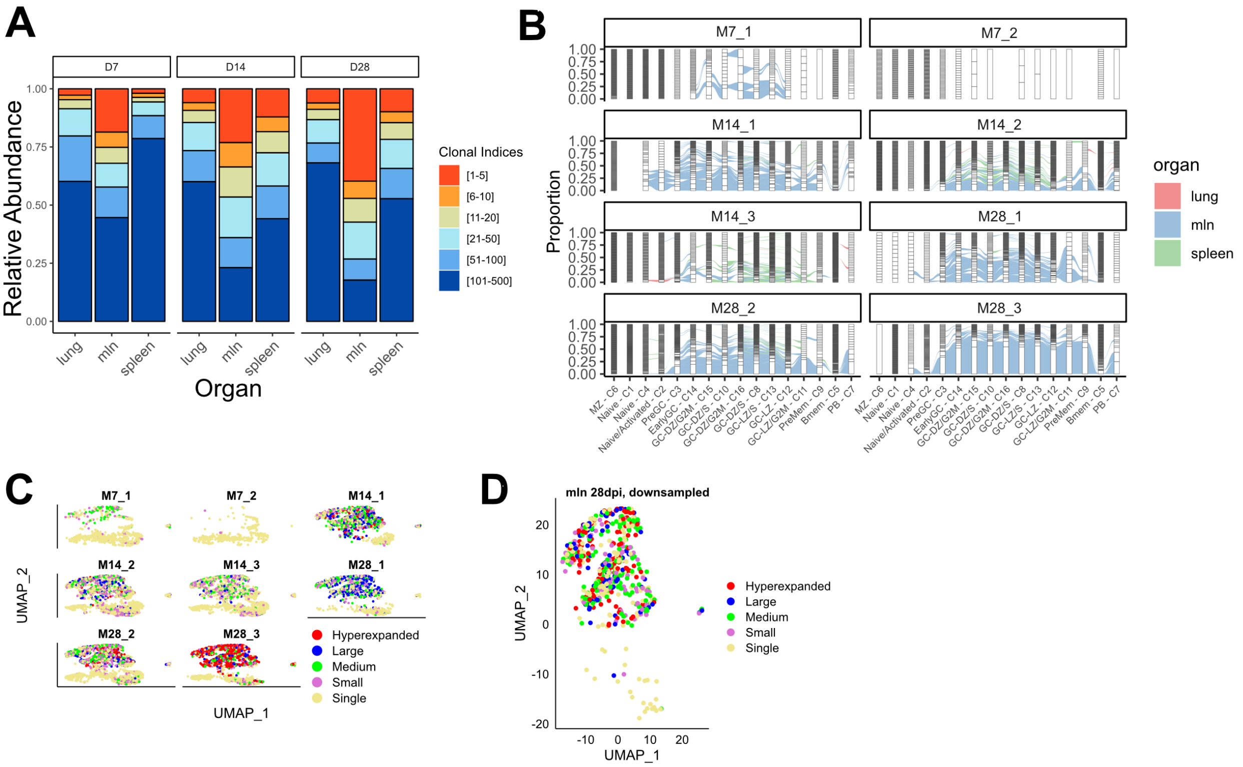

Fig S5. Clonal expansion and clonal sharing between different organs and cell populations, Related to Fig 5.

A) Graph showing clonal expansion for each organ, divided by dpi. Clones are order by their abundance for each organ and dpi and color indicates the repertoire space occupied by the top X clones as shown in figure legend.

B) Alluvial plot showing clonal sharing between clusters and organs in different mice. Each cluster is divided in several bars, representing individual clones, and the height of each represents the proportion of the cluster occupied by that clone. Connecting lines indicate sharing of clone with colors indicating the organ.

C) UMAP plot of infected cells divided by mouse number, colored by clonal expansion status.

D) UMAP plot of infected cells for mln at 28dpi, downsampled to have the same cell number as spleen 28dpi. Clones were reassigned and cells colored by clonal expansion status.

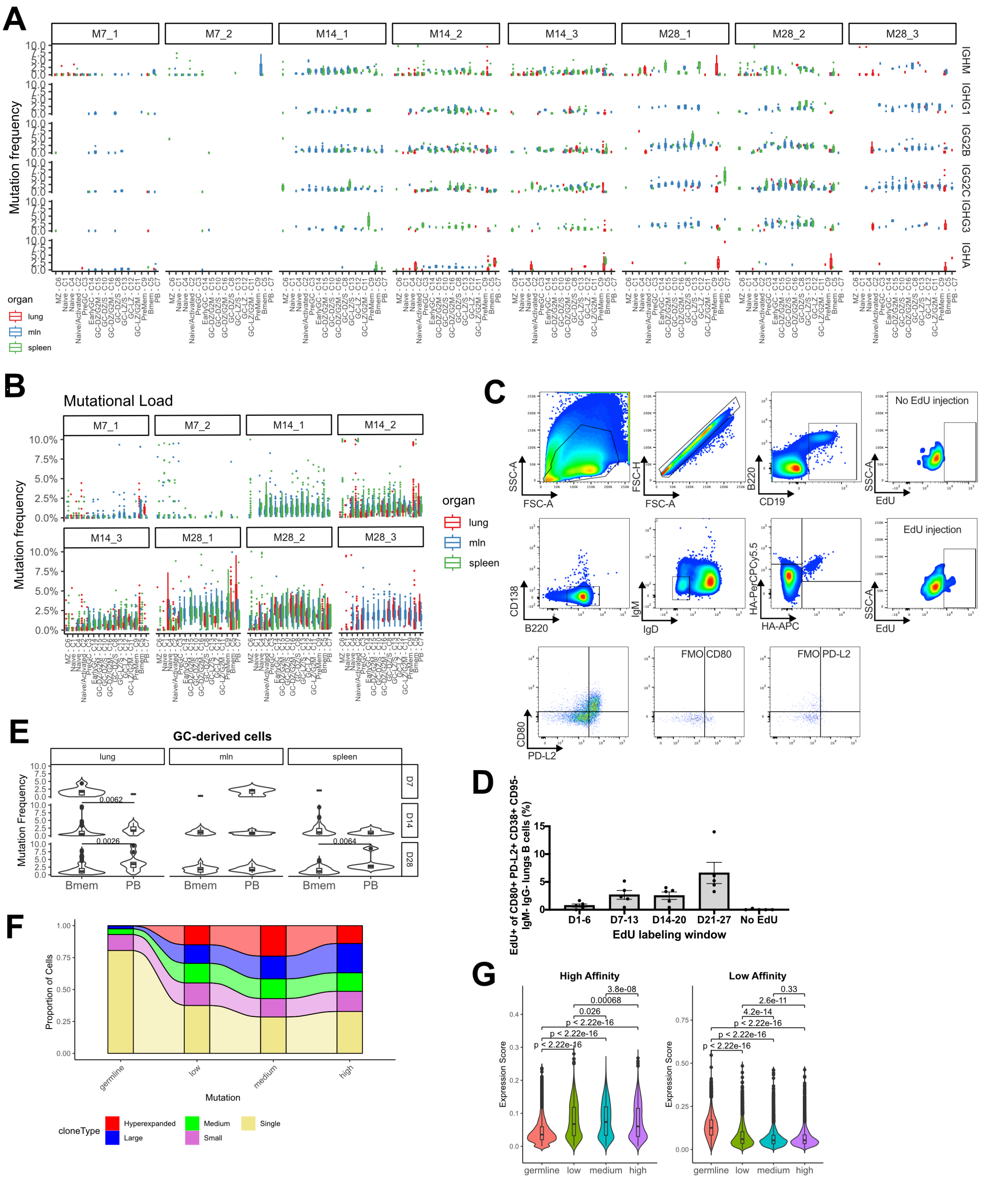

Fig S6. Differentiation and mutation frequency of B cell populations in different organs, Related to Fig 6.

A) Graph showing Vh gene mutation frequency for each cluster, divided by mouse, organ and isotype. Data are presented as the median and interquartile range.

B) Graph showing Vh gene mutation frequency for each cluster, divided by mouse and organ. Data are presented as the median and interquartile range.

C) Gating strategy for Fig 6F to identify EdU+ cells and CD80+ PDL2+ cells
D) Mice were infected with PR8 and injected with EdU at the indicated time windows. At day 35, mice were sacrificed and lungs were subjected to flow cytometry. Shown is the frequency of EdU+ cells among CD80+ PDL2+ switched memory cell population. Data are presented as mean±SEM.
E) Violin Plot comparing mutation frequency of GC-derived and PB were divided by organ and dpi. Data are presented as the median and interquartile range.Two-way ANOVA with Tukey´s post test Only significant values are shown.
F) Alluvial plot showing proportion of mutated cells, depending on clonal family size.
G) Violin Plot showing High and Low affinity gene expression scores as defined by Shinnakasu et al, stratified by BCR mutation. Data are presented as the median and interquartile range. One-Way ANOVA followed by Tukey post-hoc test.

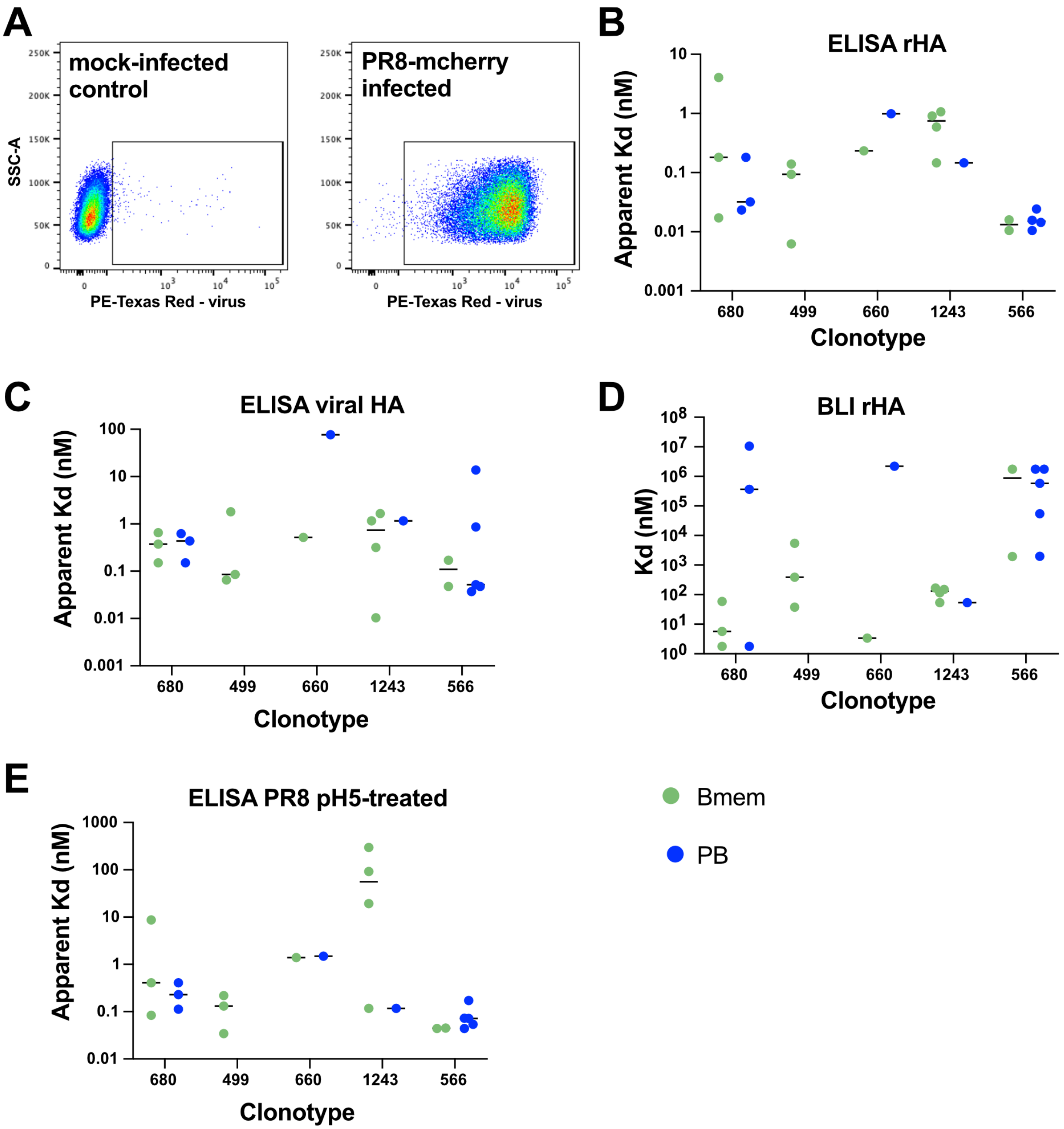

Fig S7. Affinity of expressed mAbs, Related to Fig 7.

A) Gating strategy for Fig. 7B

B) Apparent K_D value of the expressed mAbs, as measured by ELISA on recombinant HA. Bmem in green and PB in blue, divided by clonotype. No significant differences by Two-Way ANOVA followed by Tukey post-hoc test. Line represent the mean.

C) Apparent K_D value of the expressed mAbs, as measured by ELISA on viral HA. Bmem in green and PB in blue, divided by clonotype. No significant differences by Two-Way ANOVA followed by Tukey post-hoc test. Line represent the mean.

D) K_D value of the expressed mAbs, as measured by BLI on recombinant HA. Bmem in green and PB in blue, divided by clonotype. No significant differences by Two-Way ANOVA followed by Tukey post-hoc test. Line represent the mean.

E) Apparent K_D value of the expressed mAbs, as measured by ELISA on PR8 virus, treated with pH5 to expose criptic epitopes. Bmem in green and PB in blue, divided by clonotype. No significant differences by Two-Way ANOVA followed by Tukey post-hoc test. Lines represent the mean.
